# ShcA Protects against Epithelial–Mesenchymal Transition through Compartmentalized Inhibition of TGF-β-Induced Smad Activation

**DOI:** 10.1371/journal.pbio.1002325

**Published:** 2015-12-17

**Authors:** Baby Periyanayaki Muthusamy, Erine H. Budi, Yoko Katsuno, Matthew K. Lee, Susan M. Smith, Amer M. Mirza, Rosemary J. Akhurst, Rik Derynck

**Affiliations:** 1 Departments of Cell and Tissue Biology, University of California, San Francisco, San Francisco, California, United States of America; 2 Eli and Edythe Broad Center of Regeneration Medicine and Stem Cell Research, University of California, San Francisco, San Francisco, California, United States of America; 3 Center for Craniofacial Molecular Biology, Ostrow School of Dentistry, University of Southern California, Los Angeles, California, United States of America; 4 XOMA Corp., Berkeley, California, United States of America; 5 Department of Anatomy, University of California, San Francisco, San Francisco, California, United States of America; 6 Helen Diller Comprehensive Cancer Center, University of California, San Francisco, San Francisco, California, United States of America; University of Zurich, SWITZERLAND

## Abstract

Epithelial–mesenchymal transition (EMT) is a normal cell differentiation event during development and contributes pathologically to carcinoma and fibrosis progression. EMT often associates with increased transforming growth factor-β (TGF-β) signaling, and TGF-β drives EMT, in part through Smad-mediated reprogramming of gene expression. TGF-β also activates the Erk MAPK pathway through recruitment and Tyr phosphorylation of the adaptor protein ShcA by the activated TGF-β type I receptor. We found that ShcA protects the epithelial integrity of nontransformed cells against EMT by repressing TGF-β-induced, Smad-mediated gene expression. p52ShcA competed with Smad3 for TGF-β receptor binding, and down-regulation of ShcA expression enhanced autocrine TGF-β/Smad signaling and target gene expression, whereas increased p52ShcA expression resulted in decreased Smad3 binding to the TGF-β receptor, decreased Smad3 activation, and increased Erk MAPK and Akt signaling. Furthermore, p52ShcA sequestered TGF-β receptor complexes to caveolin-associated membrane compartments, and reducing ShcA expression enhanced the receptor localization in clathrin-associated membrane compartments that enable Smad activation. Consequently, silencing ShcA expression induced EMT, with increased cell migration, invasion, and dissemination, and increased stem cell generation and mammosphere formation, dependent upon autocrine TGF-β signaling. These findings position ShcA as a determinant of the epithelial phenotype by repressing TGF-β-induced Smad activation through differential partitioning of receptor complexes at the cell surface.

## Abstract 2

The adaptor protein ShcA protects epithelial cells from transitioning toward a mesenchymal phenotype by controlling partitioning of the TGF-β receptor and repressing downstream Smad2/3 activation.

## Author Summary

TGF-β family proteins control cell differentiation and various cell functions. Increased TGF-β signaling, acting through heteromeric receptor complexes, contributes to carcinoma progression and fibrosis. TGF-β drives epithelial–mesenchymal transdifferentiation (EMT), which enables cell migration and invasion. Upon TGF-β binding, “type I” receptors activate, through phosphorylation, Smad2 and Smad3 that control target gene transcription. In EMT, Smad complexes activate the expression of EMT “master” transcription factors and cooperate with these to repress the epithelial phenotype and activate mesenchymal gene expression. TGF-β receptors also activate Erk MAPK signaling, involving association of the adaptor protein ShcA and Tyr phosphorylation of ShcA by type I receptors. We now show that the predominant ShcA isoform, p52ShcA, competes with Smad2/3 for binding to type I TGF-β receptors, thus repressing Smad2/3 activation in response to TGF-β and localizing the receptors to caveolar compartments. Consequently, decreased ShcA expression enhanced TGF-β receptor localization in clathrin compartments and autocrine Smad2/3 signaling, repressed the epithelial phenotype, and promoted EMT. The changes following decreased ShcA expression resulted in increased cell migration and invasion, as well as increased stem cell generation, dependent upon autocrine TGF-β signaling. These findings position ShcA as a determinant of the epithelial phenotype by repressing TGF-β-induced Smad activation through differential partitioning of receptor complexes at the cell surface.

## Introduction

Shc proteins are intracellular adaptor proteins that relay signals from membrane-associated receptors, including receptor tyrosine (Tyr) kinases (RTKs), cytokine receptors and integrins. They interact with phospho-Tyr residues through their N-terminal PTB domain and C-terminal SH2 domain and enable Tyr kinases to phosphorylate Shc on three Tyr residues in a central CH1 domain, thus facilitating activation of the Ras/Erk mitogen-activated protein kinase (MAPK) pathway in response to extracellular ligands [1,2]. Among the four mammalian Shc proteins, ShcA is widely expressed and generated as three isoforms, p66, p52, and p46, through differential start codon usage and splicing. ShcA is well studied as a signaling mediator of membrane-associated Tyr kinases leading to Erk MAPK activation [1,2], although it also plays a role in activation of PI3K-Akt signaling [2–4] and controls cytoskeletal changes [2,5]. Targeted inactivation of ShcA expression does not prevent growth factor-induced Erk MAPK activation but confers an impaired sensitivity to growth factors and an attenuated Erk MAPK activation response [6]. Since nonchordate metazoans lack some or all Tyrs that are phosphorylated [7,8], Shc proteins may also exert functions independent of Tyr phosphorylation. ShcA is additionally controlled by serine (Ser) and threonine (Thr) phosphorylation, which regulates protein interactions, Shc activities in lipid metabolism, endocytosis and small GTPase regulation, e.g., following protein kinase C activation [9,10] and responses to epidermal growth factor (EGF) receptor activation [11]. p52ShcA also plays a role in transforming growth factor-β (TGF-β) signaling, which is not initiated by Tyr kinases [12].

TGF-β family proteins control cell differentiation and various functions in metazoans. As secreted dimers, TGF-β and TGF-β-related proteins activate intracellular signaling through a cell surface complex of two type II and two type I receptor kinases. Upon ligand binding, the type II receptors phosphorylate the type I receptors that then activate their signaling effectors, the Smads, through C-terminal phosphorylation on two Sers. Thus, TGF-β induces the type I receptor TβRI to activate Smad2 and/or Smad3, which then dissociate from the receptor complexes and form trimers of two receptor-activated Smads and one Smad4. These then cooperate with DNA binding transcription factors and coregulators to activate or repress TGF-β target gene expression [13–15]. In addition to the Smad-mediated changes in transcription, the TGF-β receptors also activate Erk, c-Jun N-terminal kinase (JNK), and p38 MAPK signaling, as well as Rho and PI3K-Akt-TOR signaling, albeit to a lower extent than RTKs [16–18]. Their activation in response to TGF-β may relate to the dual kinase specificity of the TGF-β receptor [12,19–21], which, as is seen with other dual specificity kinases [22], confers Tyr phosphorylation that is much weaker than Ser/Thr phosphorylation [12,21]. TGF-β induces TβRI phosphorylation on Tyr, and TGF-β-induced activation of Erk MAPK signaling results from TGF-β-induced recruitment of p52ShcA to TβRI, enabling TβRI to phosphorylate p52ShcA on Tyr, and more prominently on Ser [12].

At several stages during development, dependent on the microenvironment, epithelial cells repress their differentiation state, resulting in loss of epithelial junction and polarity complexes, and redirect their gene expression and phenotype to transition into motile mesenchymal cells [23,24]. This reversible transdifferentiation process is commonly called epithelial–mesenchymal transition (EMT) and is potently induced by activation of TGF-β signaling, in cooperation with other signaling mediators [24–27]. Postnatally, EMT is recapitulated in fibrosis [28,29] and in cancer progression, where it directs carcinoma cell invasion and correlates with cancer stem cell properties. In both pathological contexts, EMT has been functionally linked with increased TGF-β signaling [25,30–32]. In TGF-β-induced EMT, the activated Smads direct the expression of “master” transcription factors, such as Snail, ZEB, and Twist, and then cooperate with these transcription factors to regulate the expression of target genes [24]. While Akt activation is required for completion of TGF-β-induced EMT, with mTOR complex 2 being essential for the cytoskeletal reorganization and motility [33,34], the roles of the MAPK pathways in TGF-β-induced EMT have been less defined, although Erk MAPK signaling has been implicated in EMT responses [26,35].

Aiming to better understand the roles of TGF-β signaling in EMT, we down-regulated the expression of ShcA in epithelial cells. These cells underwent EMT spontaneously, thus enhancing their motility and invasion, and the generation of epithelial stem cells, dependent on autocrine TGF-β-induced Smad signaling. Our results show that ShcA protects epithelial cells against EMT through suppression of TGF-β-induced Smad signaling. This inhibition is achieved through direct competition of ShcA with Smad3 for binding to the TβRI, and, consequently, changes in the partitioning of ShcA-interacting TGF-β receptor complexes and receptor complexes that enable TGF-β-induced Smad activation.

## Results

### Decreasing ShcA Expression Promotes Transition from Epithelial to Mesenchymal Phenotype

**Fig 1:**
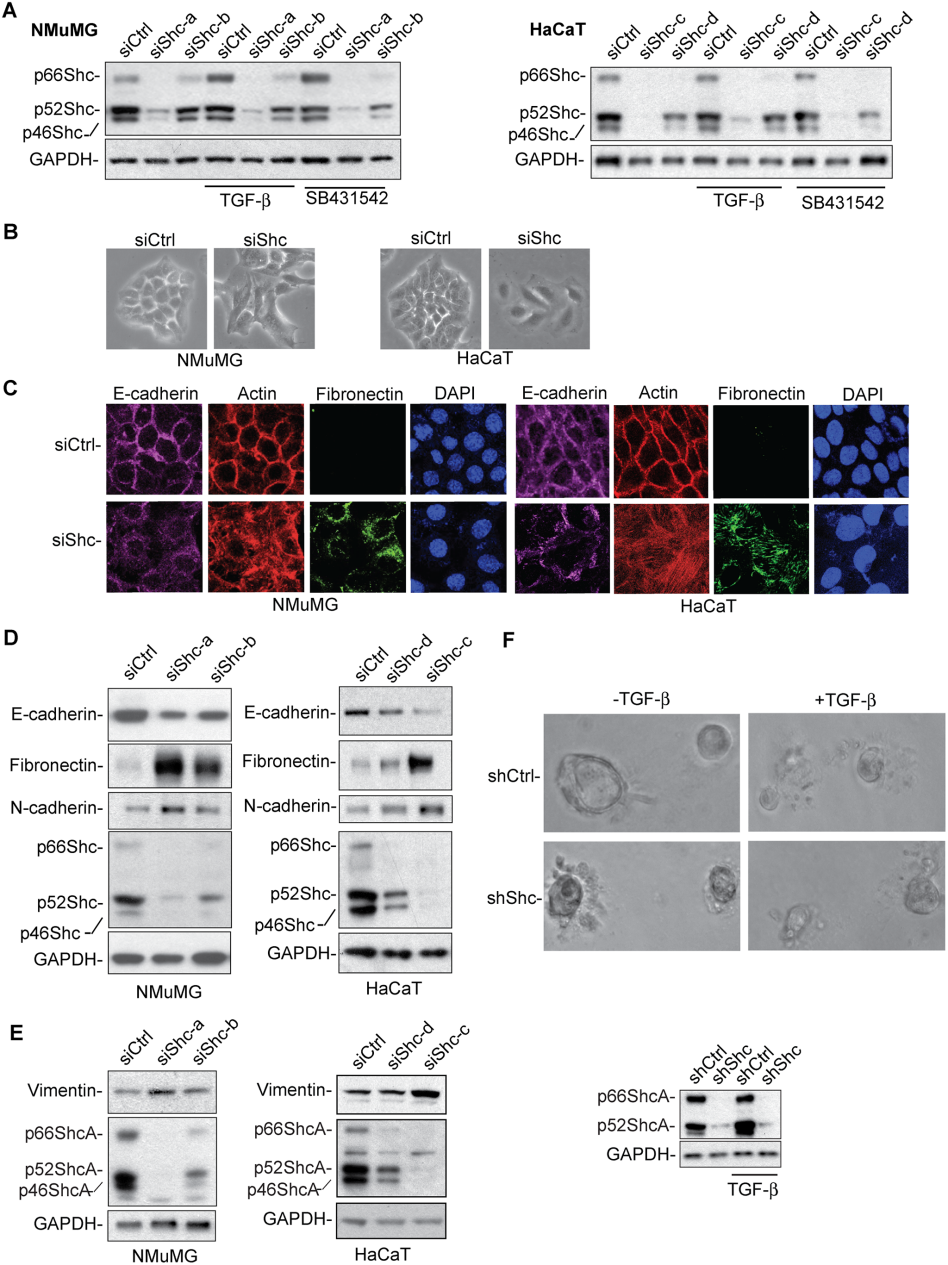
Decreased ShcA expression promotes EMT in NMuMG and HaCaT cells. (A) ShcA levels, assessed by immunoblotting, in NMuMG and HaCaT cells transfected with ShcA or control siRNA. siShc-a and siShc-b are ShcA siRNAs used in NMuMG cells, and siShc-c and siShc-d are used in HaCaT cells. Glyceraldehyde-3-phosphate dehydrogenase (GAPDH) immunoblotting serves as loading control. (B) Phase contrast microscopy of NMuMG and HaCaT cells transfected with ShcA siRNA (siSchc-a for NMuMG cells, siShc-c for HaCaT cells) or control siRNA. (C) F-actin staining, and E-cadherin or fibronectin immunostaining of NMuMG and HaCaT cells transfected with control siRNA or ShcA siRNA (siShc-a for NMuMG cells, siShc-c for HaCaT cells). Nuclei are visualized by 4’,6-diamidino-phenol-indole (DAPI) staining. (D, E) E-cadherin, fibronectin, N-cadherin, and vimentin expression, shown by immunoblotting, in NMuMG and HaCaT cells, transfected with control or ShcA siRNA (siShc-a and siShc-b for NMuMG cells, siShc-c and siShc-d for HaCaT cells). Down-regulation of ShcA expression upon ShcA siRNA transfection confers decreased E-cadherin expression and increased expression of fibronectin, N-cadherin and vimentin. GAPDH immunoblotting serves as loading control. (F) Phase contrast images of 3-D cultures of NMuMG cells, infected with a lentiviral vector expressing control shRNA or ShcA shRNA, in Matrigel in the absence or presence of added TGF-β. The immunoblot shows the ShcA expression and GAPDH as loading control in the cells used for 3-D culture. All experiments were reproducibly repeated at least three times. Supplemental data are shown in S1 Fig.

To address the role of ShcA in the epithelial phenotype and EMT, we used mouse mammary NMuMG cells and human HaCaT skin keratinocytes as model systems, which like most other cells predominantly express the p52ShcA isoform (Fig 1A). In these cells, which transition into a mesenchymal phenotype in response to TGF-β [34,36,37], we down-regulated ShcA expression by transfecting the cells with ShcA-specific small interfering RNAs (siRNAs) (Fig 1A; S1A Fig). ShcA mRNA expression was at its best decreased to 15%, as assessed by qRT-PCR, depending on the sequence of the siRNA (S1B Fig).

Compared to cells transfected with control siRNA, cells with down-regulated ShcA expression had a less cuboidal epithelial phenotype and a more elongated and spread out phenotype (Fig 1B), suggesting EMT. The EMT phenotype was supported by decreased immunostaining and diffuse mislocalization of E-cadherin at cell contacts, shown by confocal immunofluorescence (Fig 1C). Furthermore, the actin filaments were no longer cortically organized, and the cells showed prominent fibronectin immunostaining that was not apparent in epithelial cells that were transfected with control siRNA (Fig 1C). These changes upon down-regulation of ShcA expression were similar in NMuMG and HaCaT cells (Fig 1B and 1C). That down-regulation of ShcA expression resulted in EMT was also supported by decreased E-cadherin expression and increased fibronectin, N-cadherin and vimentin expression that characterize EMT. Also these changes were apparent in both NMuMG and HaCaT cells, when compared with cells transfected with control siRNA (Fig 1D and 1E; S1C and S1D Fig).

The EMT phenotype resulting from decreased ShcA expression could be reversed following reintroduction of p52ShcA expression. HaCaT cells, selected to express an shRNA that targets the ShcA 3’ untranslated region from a lentiviral vector, had decreased ShcA expression (S1E Fig) and showed the expected EMT phenotype, as apparent by cell morphology (S1E Fig, top), decreased E-cadherin and increased fibronectin expression (S1G and S1H Fig). Reintroducing p52ShcA expression using a vector that was not targeted by the shRNA (S1E Fig) reverted the cells to an epithelial phenotype with increased E-cadherin and decreased fibronectin expression (S1G and S1H Fig).

Finally, NMuMG cells can form acini-like structures when allowed to grow three-dimensionally in Matrigel, and inducing EMT in response to TGF-β impairs the integrity of these structures due to cell dispersal [38,39]. Whereas NMuMG cells transfected with control siRNA formed such smooth-edged spheres in Matrigel, cells with decreased ShcA expression were unable to do so and showed cell dispersal, similarly to the effect of adding TGF-β (Fig 1F).

Based on these observations, we conclude that decreasing ShcA expression confers a transition from an epithelial to a mesenchymal phenotype, and, therefore, that ShcA plays a role in stabilizing the epithelial phenotype.

### ShcA Expression Controls EMT-Associated Cell Behavior

**Fig 2:**
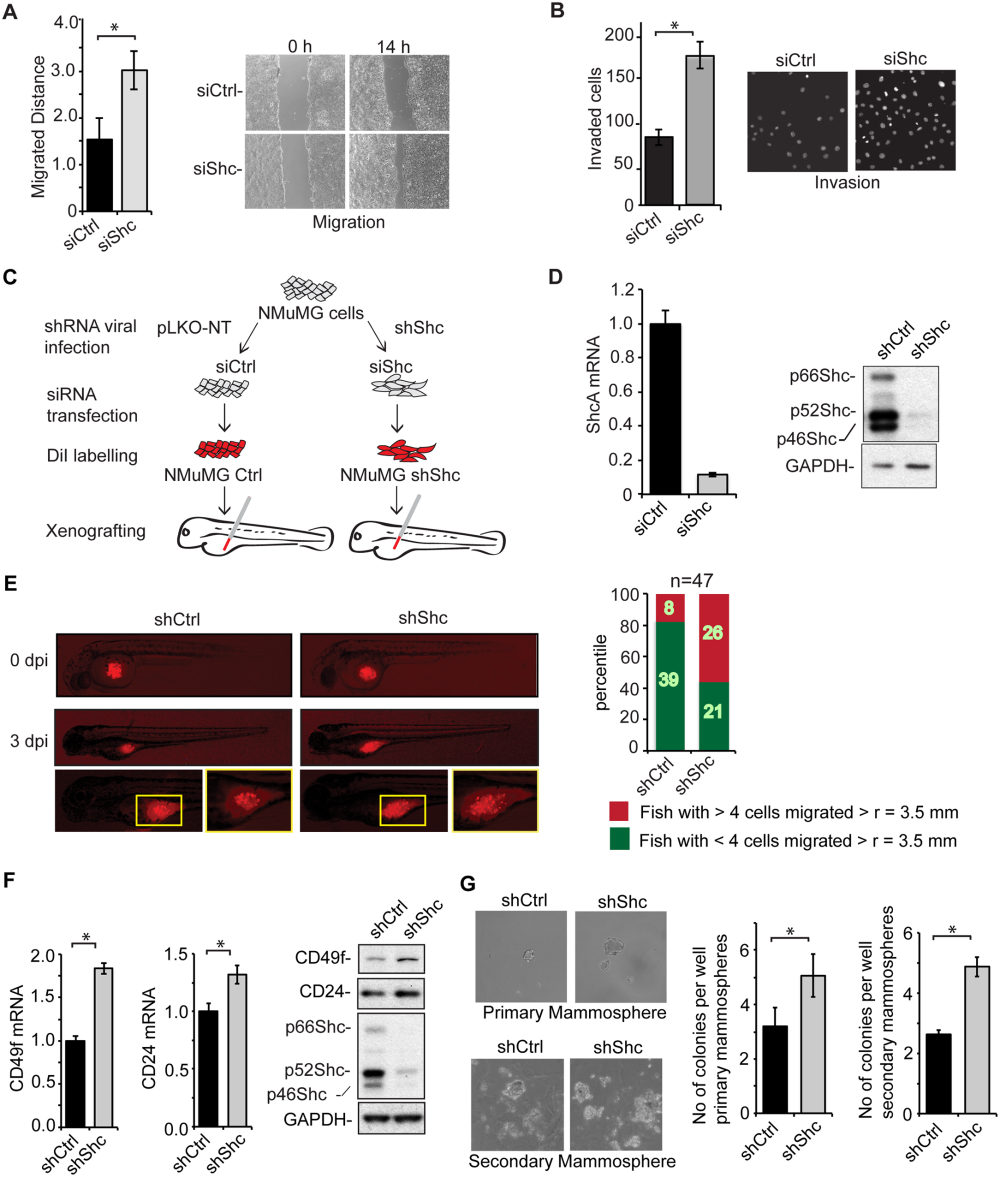
ShcA expression controls EMT-associated cell behavior. (A) Migration of NMuMG cells, assessed using a monolayer scratch assay, and visualized and quantified by phase contrast microscopy at 0 and 14 h. Cells were transfected with control siRNA or ShcA siRNA (siShc-a). Error bars show standard error of the mean, based on three independent experiments. (B) Invasion of NMuMG cells through Matrigel in Transwell assays. Cells were transfected with control siRNA or ShcA siRNA (siShc-a). Cells at the bottom of the membrane were visualized using DAPI and counted after 36 h in four fields in triplicate. Error bars show standard error of the mean, based on three independent experiments. (C) Schematic diagram of the xenograft injections of NMuMG cells into zebrafish embryos. Cells were lentivirally infected with control vector or a vector expressing shRNA to ShcA, then transfected with control siRNA or ShcA siRNA (siShc-a), and then labeled with DiI. The DiI-labeled cells were injected into the yolk sac of zebrafish embryos, and their dissemination visualized after 3 d by fluorescence microscopy. (D) Decreased expression of ShcA mRNA, assessed by qRT-PCR and normalized to RPL19 mRNA (left), and protein, assessed by immunoblotting (right), in NMuMG cells lentivirally expressing shRNA and transfected with ShcA siRNA. Error bars show standard error of the mean, based on three independent experiments. (E) Localization of diI-labeled NMuMG cells, either control cells or cells with decreased ShcA expression, in zebrafish, immediately after injection (0 dpi) or at day 3 after injection (3 dpi). Each bar of the histogram shows the proportion of the total sample of injected fish (*n* = 47 for each group) with at least 4 DiI-labeled cells that migrated > 3.5 mm away from the injection site (red), or with no or less than 4 cells that migrated more than 3.5 mm from the injection site (green). (F) Expression of CD49f mRNA and CD24 mRNA, quantified by qRT-PCR and normalized to RPL19 mRNA, and CD49f and CD24 protein expression, assessed by immunoblotting, in cells lentivirally infected to express control or ShcA shRNA. Error bars show standard error of the mean, based on three independent experiments. (G) NMuMG cells were lentivirally infected to express control or ShcA shRNA and mammosphere formation efficiencies were evaluated. Primary mammospheres were dissociated and then re-seeded in suspension as single cells to give rise to secondary mammospheres. Error bars show standard error of the mean, based on three independent experiments. All experiments were reproducibly repeated at least three times. Statistical analyses were performed using two-tailed two-sample unequal variance *t* test. *, *p* < 0.05. Supplemental data are shown in S2 Fig and S1 Data.

The epithelial to mesenchymal phenotype transition is marked by increased cell motility, which is often the basis for increased invasiveness of cells that have undergone EMT, e.g., in cancer progression [23,24]. We therefore evaluated whether decreased ShcA expression gave rise to cells with enhanced motility and invasion. As shown in Fig 2A, down-regulation of ShcA expression increased cell motility, assessed in a cell monolayer wounding assay. Additionally, the cells showed increased invasion in Transwell assays that score the number of cells that invaded through basement membrane (Fig 2B).

To examine the effect of decreased ShcA expression on cell behavior in vivo, we transplanted DiI-labeled cells into the yolk sac of zebrafish embryos (Fig 2C). Recent studies have shown that cancer cell dissemination in such zebrafish xenograft assays correlates with cancer cell behavior in mouse models of metastases [40]. DiI-labeled NMuMG cells with down-regulated ShcA expression (Fig 2D; S2A Fig) and control NMuMG cells were injected into the yolk sac of zebrafish embryos (Fig 2C), and the dissemination of the cells was visualized by fluorescence microscopy over 60–84 h. Down-regulation of ShcA expression resulted in increased dissemination from the site of injection, scored at 72 h after injection (Fig 2E), which is consistent with their increased cell motility and invasion (Fig 2A and 2B).

Partial or complete loss of epithelial phenotype and EMT can lead to acquisition of stem cell properties in normal and transformed mammary epithelial cells [31,41]. Mammary stem cells that have the ability to self-renew and reconstitute mammary glands can form single cell–derived mammospheres when cultured in suspension [42]. Additionally, mouse mammary epithelial or carcinoma stem cells express increased levels of CD49f and moderately increased CD24 levels, when compared to cells lacking self-renewal and gland-reconstituting capacities [43,44]. Accordingly, we found that decreasing ShcA expression resulted in increased CD49f expression and a modest increase in CD24 expression (Fig 2F; S2B Fig). Additionally, down-regulation of ShcA expression conferred increased formation of single cell–derived mammospheres (Fig 2G), indicative of the increased number of self-renewing stem cells in the population. These results correlate the EMT phenotype, resulting from decreased ShcA expression, with stem cell properties in culture, and suggest that ShcA expression may control cancer stem cell generation and function.

### ShcA Does Not Modulate EMT through the Erk MAPK Pathway

**Fig 3:**
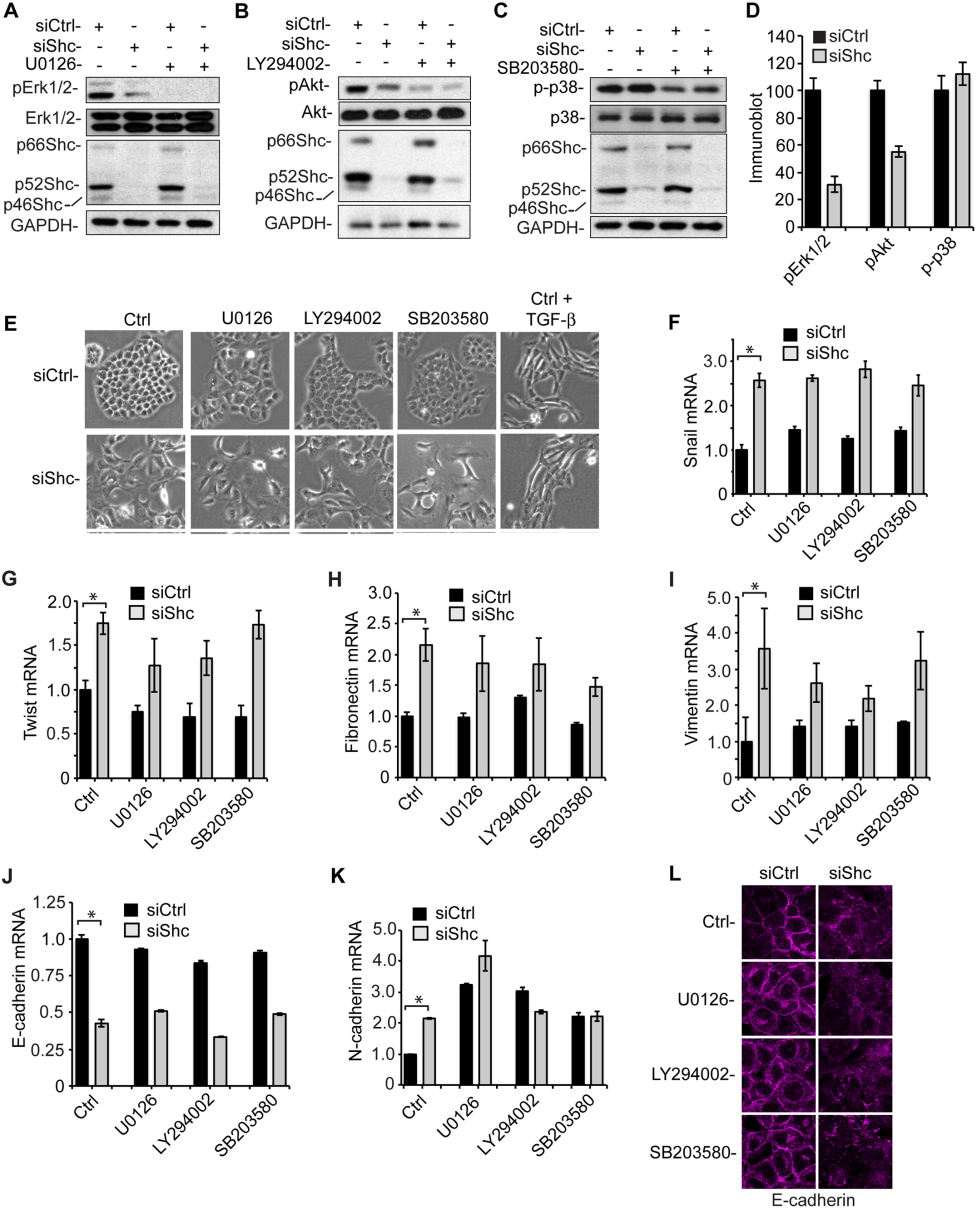
Effects of MEK1/2, PI3K, and p38 MAPK inhibition on the EMT phenotype of NMuMG cells without or with down-regulated ShcA expression. (A–D) NMuMG cells transfected with control siRNA or ShcA siRNA (siShc-a) were treated or not with the MEK1/2 inhibitor U0126 (A), the PI3K inhibitor LY294002 (B), or the p38 MAPK inhibitor SB203580 (C) for 36 h, and the level of signaling pathway activation was assessed by immunoblotting for pErk1/2, with Erk1/2 as control (A), pAkt, with Akt as control (B), pp38 MAPK, with p38 MAPK as control (C). Immunoblotting for ShcA revealed the down-regulation of ShcA expression using siRNA, and GAPDH immunoblotting served as loading control. In (D), the immunoblots of pErk1/2, pAkt, and pp38MAPK in NMuMG cells transfected with ShcA siRNA were quantified by densitometry, relative to the levels in cells transfected with control siRNA. The graphs show averaged values of three independent experiments, with error bars indicating standard errors. (E–L) Cell morphology and gene expression of NMuMG cells transfected with ShcA siRNA (siShc-a), treated for 36 h with the MEK1/2 inhibitor U0126, the PI3K inhibitor LY294002 or the p38 MAPK inhibitor SB203580. In (E), the cell morphology was assessed by phase contrast microscopy, whereas Snail (F), Twist (G), fibronectin (H), vimentin (I), E-cadherin (J) and N-cadherin (K) mRNA were quantified by qRT-PCR and normalized to RPL19 mRNA, and in (L) E-cadherin was visualized by immunofluorescence. The graphs show averaged values of three independent experiments, with error bars indicating standard errors, based on three experiments. Statistical analyses were performed using two-tailed two-sample unequal variance *t* test. *, *p* < 0.05. Supplemental data are shown in S3 Fig and S1 Data.

ShcA acts as an adaptor that facilitates Erk MAPK pathway in response to various ligands, as has been best studied in the context of growth factor-induced Tyr kinase receptor signaling [1,2]. Cells lacking ShcA expression, as a result of targeted gene inactivation, show attenuated growth factor-induced Erk MAPK activation [6]. Accordingly, down-regulation of ShcA expression in NMuMG cells reduced the level of Erk MAPK activation under our cell culture conditions with serum (Fig 3A and 3D). ShcA down-regulation also exerted a milder decrease in basal Akt activation (Fig 3B and 3D), consistent with the proposed role of Shc in insulin- and growth factor-induced PI3K activation [2,3,45]. No effect was seen on the basal level of p38 MAPK activation (Fig 3C and 3D).

To assess whether the EMT resulting from decreased ShcA expression was due to decreased Erk MAPK pathway activity, we evaluated the effect of U0126, a MEK1/2 inhibitor that prevents Erk MAPK activation (Fig 3A), on cell morphology. U0126 did not induce the phenotypic changes that are apparent when ShcA expression is down-regulated (Fig 3E). U0126 also did not induce Snail, Twist, fibronectin or vimentin expression, or repress E-cadherin expression, as is seen in response to silencing ShcA expression (Fig 3F–3J). However, inhibition of MEK1/2 activity resulted in a somewhat increased N-cadherin expression (Fig 3K), and slightly repressed Twist expression (Fig 3G). Furthermore, U0126 slightly destabilized the junctional localization of E-cadherin that was revealed by immunofluorescence, with further destabilization upon down-regulation of ShcA expression (Fig 3L). Similarly to NMuMG cells, treatment of HaCaT cells with U0126 did not induce a change in morphology that resembled the effect of down-regulation of ShcA expression (S3A Fig), and did not induce the expression of the Snail-related transcription factor Slug (S3B Fig), which, similarly to Snail in NMuMG, promotes EMT of HaCaT cells [46]. These data suggest that inhibition of the MEK1/2-Erk MAPK pathway does not account for a loss of epithelial morphology, and argue that EMT resulting from ShcA down-regulation is not due to inhibition of the Erk MAPK pathway.

Considering the mild decrease in Akt activation following ShcA down-regulation, and the reported roles of Shc in growth factor-induced PI3K activation [2,3,45], we also assessed the effects of LY294002, a direct PI3K inhibitor, on the epithelial morphology. Inhibition of PI3K activity resulted in decreased Akt activation (Fig 3B), without, however, inducing an EMT-like morphology (Fig 3E) or Snail or Twist mRNA expression in control epithelial cells, nor did it significantly affect the expression of fibronectin, vimentin or E-cadherin mRNA (Fig 3F–3J). These data suggest that the EMT-like phenotype following down-regulation of ShcA expression does not result from decreased Akt activation. Finally, inhibition of p38 MAPK using SB203580 did not affect the epithelial morphology in control cells, or the EMT phenotype in cells with down-regulated ShcA expression (Fig 3E), and had no major effects on the expression of EMT marker genes (Fig 3F–3K). Similar results were obtained in HaCaT cells (S3A and S3B Fig).

### Decreased ShcA Expression Promotes EMT through Autocrine TGF-β Signaling

**Fig 4:**
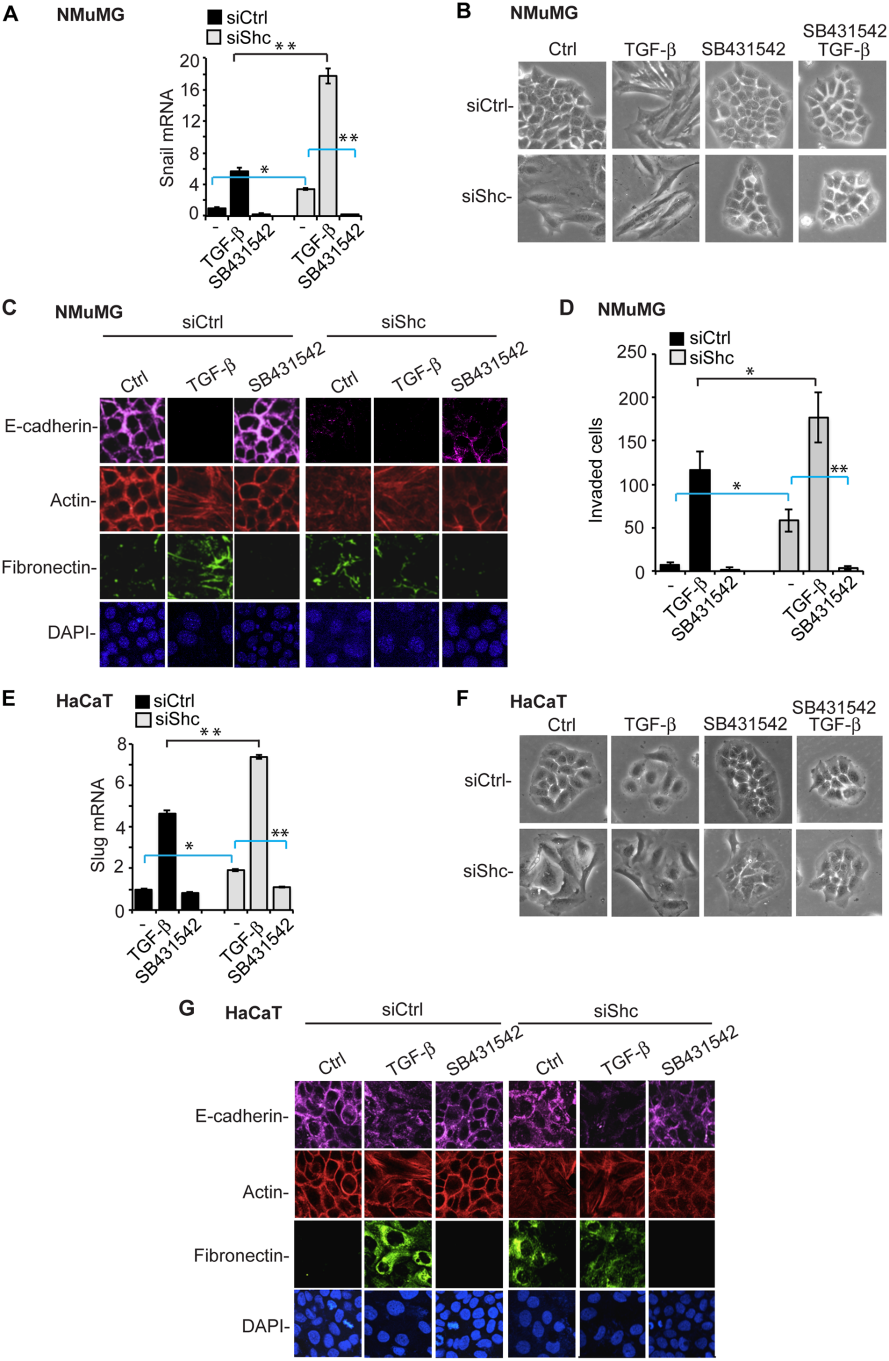
The TβRI kinase activity is required for EMT in epithelial cells with down-regulated ShcA expression. (A–D) The TβRI kinase inhibitor SB431542 inhibits Snail mRNA expression, assessed by qRT-PCR and normalized to RPL19 mRNA (A), EMT morphology, assessed by phase contrast microscopy (B), downregulation of cortical E-cadherin, redistribution of actin, and activation of fibronectin expression, assessed by immunofluorescence (C), and cell invasion through Matrigel, assessed as in Fig 2B (D), in control NMuMG cells treated or not with TGF-β for 48 h, and NMuMG cells transfected with ShcA siRNA (siShc-a). (E–G) SB431542 inhibits Slug mRNA expression, assessed by qRT-PCR and normalized to RPL19 mRNA (E), EMT morphology, assessed by phase contrast microscopy (F), and the down-regulation of cortical E-cadherin, redistribution of actin and activation of fibronectin expression, assessed by immunofluorescence (G), in control HaCaT cells treated or not with TGF-β for 72 h, or HaCaT cells transfected with ShcA siRNA (siShc-c). DAPI staining visualized the nuclei. The graphs (A, D, E) show averaged values of three independent experiments, with error bars indicating standard errors, based on three experiments. Statistical analyses were performed using two-tailed two-sample unequal variance *t* test. *, *p* < 0.05; **, *p* < 0.002. Supplemental data are shown in S4 Fig and S1 Data.

To define the mechanism that accounts for the EMT in response to down-regulation of ShcA expression, we treated the cells with SB431542, a specific inhibitor of the TGF-β/activin type I receptor kinases that prevents TGF-β-induced Smad2/3 activation [47]. As expected, SB431542 blocked the TGF-β-induced activation of Snail mRNA expression (Fig 4A; S4A Fig) and the TGF-β-induced transition of NMuMG cells into an elongated cell phenotype (Fig 4B). In untreated NMuMG cells with down-regulated ShcA expression, SB431542 strongly decreased the level of Snail mRNA to a level below the Snail mRNA expression in NMuMG cells with control siRNA (Fig 4A; S4A Fig). A similar inhibition was observed when cells were treated with LY2109761, another TGF-β/activin type I receptor kinase inhibitor [48] (S4B Fig). Furthermore, these cells reverted to an epithelial phenotype when treated with SB431542 or LY2109761. This was apparent by visual microscopic examination (Fig 4B), immunofluorescence for E-cadherin at cell contacts and cortical actin (Fig 4C), and immunoblotting, immunofluorescence, and/or mRNA expression of mesenchymal fibronectin, vimentin, and N-cadherin (Fig 4C; S4C–S4G Fig). Treatment of the cells with a neutralizing anti-TGF-β monoclonal antibody also repressed the EMT phenotype, but this repression was less complete when compared with the effects of SB431542 or LY2109761 (S4B–S4D Fig), consistent with an inability to fully block autocrine TGF-β signaling using antibodies. Furthermore, SB431542 inhibited the invasion that resulted from down-regulation of ShcA expression (Fig 4D). As in NMuMG cells, SB431542 also induced HaCaT cells with down-regulated ShcA expression to revert from the mesenchymal into an epithelial phenotype (Fig 4E–4G). Indeed, SB431542 repressed the enhanced expression of the Snail-related transcription factor Slug (Fig 4E; S4H Fig) and reverted the cells to an epithelial appearance (Fig 4F) with epithelial E-cadherin and actin staining and lack of fibronectin immunostaining (Fig 4G). These observations suggest that autocrine TGF-β signaling, to which all cells in culture are exposed, drives the observed EMT in cells with decreased ShcA expression. By extension, these data also suggest that down-regulation of ShcA expression confers increased sensitivity to autocrine TGF-β signaling.

### Down-Regulation of ShcA Expression Enhances Autocrine, TGF-β-Induced Smad Signaling

**Fig 5:**
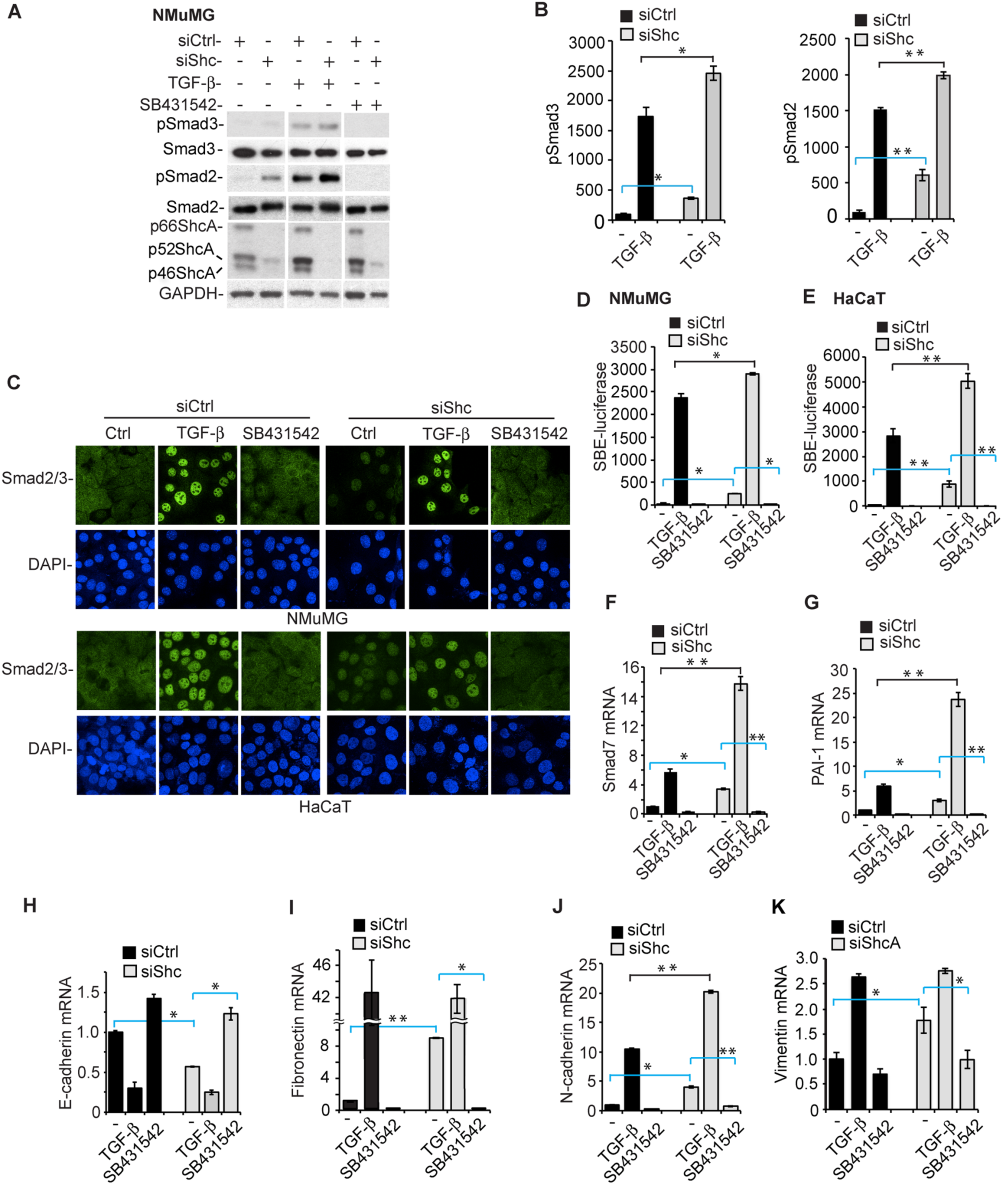
Decreasing ShcA expression enhances TGF-β-induced Smad signaling. (A) Smad3 and Smad2 activation, assessed by pSmad3 or pSmad2 immunoblotting, in NMuMG cells, transfected with control or ShcA siRNA (siShc-a), in the absence of added TGF-β or in response to 2 ng/ml TGF-β for 30 min. Note the enhanced basal pSmad3 or pSmad2 levels in cells transfected with ShcA siRNA. ShcA immunoblotting reveals decreased ShcA levels in cells transfected with ShcA siRNA, and GAPDH immunoblotting serves as loading control. (B) Quantification by densitometry of the pSmad2 or pSmad3 immunoblotting of NMuMG cells, transfected with control or ShcA siRNA (siShc-a), in the absence of added TGF-β or in response to 2 ng/ml TGF-β for 30 min. Note the enhanced basal pSmad3 or pSmad2 levels in cells transfected with ShcA siRNA. The graphs show averaged values of three independent experiments, with error bars indicating standard errors. (C) Subcellular localization of Smad2/3, assessed by immunofluorescence, in NMuMG or HaCaT cells, transfected with control or ShcA siRNA (siShc-a for NMuMG cells, siShc-c for HaCaT cells), in the absence of added TGF-β or in response to 2 ng/ml TGF-β for 60 min. (D, E) Decreasing ShcA expression, upon transfection of ShcA siRNA (siShc-a for NMuMG cells, siShc-c for HaCaT cells) but not control siRNA, enhances Smad3-mediated transcription, quantified by luciferase expression from a 4xSBE-luciferase reporter, in NMuMG (D) and HaCaT (E) cells, in the absence of or in response to 0.8 ng/ml TGF-β, or treated with SB431542, for 6 h. The expression of 4xSBE-luciferase reporter was normalized against the cotransfected Renilla-Lux reporter. (F, G) Decreasing ShcA expression using transfected siShc-a siRNA enhances expression of direct TGF-β/Smad target genes, encoding Smad7 (F) or PAI-1 (G), quantified by qRT-PCR and normalized against RPL19 mRNA, in NMuMG cells. (H–K) Decreasing ShcA expression using transfected siShc-a siRNA represses the expression of E-cadherin mRNA (H) and enhances the expression of fibronectin (I), N-cadherin (J), and vimentin (K) mRNA, quantified by qRT-PCR and normalized against RPL19 mRNA, in NMuMG cells. The graphs show averaged values of three independent experiments, with error bars indicating standard errors, based on three experiments. Statistical analyses were performed using two-tailed two-sample unequal variance *t* test. *, *p* < 0.05; **, *p* < 0.005. The error bars show standard error of the mean, based on three independent experiments. Supplemental data are shown in S5 Fig and S1 Data.

We next evaluated the effect of transfected ShcA siRNA on autocrine and TGF-β-induced activation of Smad2 and Smad3, the major signaling effector in response to TGF-β. Without adding TGF-β, NMuMG cells showed marginally detectable Smad2 and Smad3 activation, visualized by immunoblotting for C-terminally phosphorylated Smad2 or Smad3 (Fig 5A). Their basal activation was higher when ShcA expression was downregulated (Fig 5A and 5B), and blocked by SB431542 (Fig 5A), reflecting autocrine TGF-β signaling. In addition, the Smad2 and Smad3 activation in response to added TGF-β was also enhanced when ShcA expression was down-regulated (Fig 5A and 5B). Since Smad activation results in nuclear localization of Smad2 and Smad3, we examined their subcellular localization by immunofluorescence for Smad2/3 (Fig 5C) and following separation of the nuclear and cytoplasmic fractions (S5A and S5B Fig). Without adding TGF-β, cells with down-regulated ShcA expression showed a distinct level of Smad2/3 nuclear localization that was much higher than in control cells (Fig 5C; S5A and S5B Fig) and was abolished when SB431542 was added to block autocrine TGF-β signaling (Fig 5C). Adding TGF-β induced a robust nuclear translocation of Smad complexes (Fig 5C). These data suggested that decreasing ShcA expression resulted in increased basal and TGF-β-induced Smad signaling.

To better appreciate the consequence of the increased Smad3 activation, we examined the transcription from tandem Smad3-binding DNA sequences in luciferase reporter assays (Fig 5D and 5E). Transfection of ShcA siRNA, resulting in decreased ShcA expression, enhanced the basal Smad-mediated transcription in NMuMG and HaCaT cells, and increased the TGF-β-induced luciferase expression (Fig 5D and 5E; S5C and S5D Fig). The enhanced Smad activation in response to autocrine TGF-β signaling, or in response to added TGF-β, predicts that down-regulation of ShcA expression results in increased TGF-β target gene expression. This was indeed the case. Activation of Smad7 or PAI-1 mRNA expression is routinely used to monitor direct TGF-β-induced, Smad-mediated transcription activation. As shown in Fig 5F and 5G, down-regulation of ShcA expression resulted in enhanced Smad7 and PAI-1 mRNA expression, which was prevented by blocking the TGF-β-induced Smad activation using SB431542. Decreased ShcA expression also enhanced Smad7 and PAI-1 expression in response to added TGF-β.

The increased autocrine induction of TGF-β target genes may be at the basis of the spontaneous EMT of NMuMG and HaCaT cells, when ShcA expression is down-regulated. Indeed, Snail mRNA expression in NMuMG cells and Slug mRNA expression in HaCaT cells were higher when ShcA expression was down-regulated, and these increases were prevented in the presence of SB431542 (Fig 4A and 4E; S4A and S4E Fig). Similarly, Twist and ZEB1 mRNA expression were enhanced upon down-regulation of ShcA expression in NMuMG and HaCaT cells, and these increases were repressed by SB431542 (S5E–S5H Fig). With Snail directing the repression of E-cadherin expression in NMuMG cells, ShcA down-regulation resulted in lower E-cadherin mRNA expression, another hallmark of EMT in NMuMG cells, that was prevented in the presence of SB431542 (Fig 5H; S5I Fig). Finally, down-regulation of ShcA expression resulted in enhanced fibronectin, N-cadherin, and vimentin mRNA expression, which was blocked by SB431542 and therefore depended on autocrine TGF-β signaling (Fig 5I–5K; S5J Fig).

These data illustrate that decreasing ShcA expression results in enhanced autocrine TGF-β/Smad signaling, and consequently in enhanced TGF-β target gene responses, which drive or contribute to the spontaneous EMT response.

### ShcA Competes with Smad3 for Binding to TβRI

**Fig 6:**
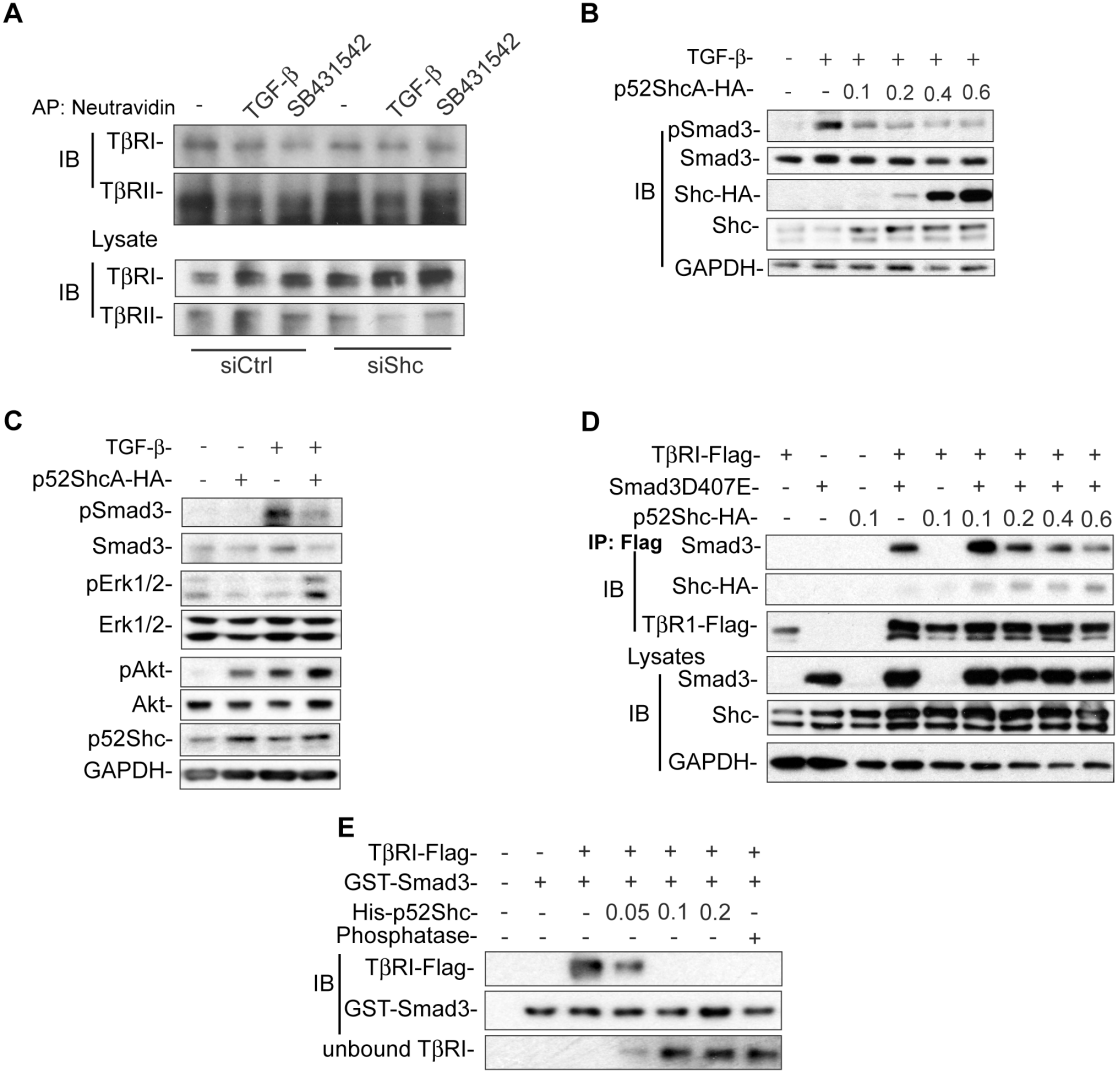
ShcA represses Smad3 activation through competition for TβRI binding. (A) Cell surface levels of TβRII and TβRI, isolated by cell surface protein biotinylation and neutravidin adsorption, in NMuMG cells transfected with control or ShcA siRNA (siShc-a) and treated or not with TGF-β or SB431542. TβRI and TβRII levels in the cell lysates were visualized by immunoblotting. (B) Increasing quantities of transfected p52ShcA expression plasmid, in μg per well, decreased the TGF-β-induced Smad3 activation, shown by immunoblotting for pSmad3, in NMuMG cells. Immunoblotting reveals the total Smad3 levels, increasing levels of transfected p52ShcA-HA, and total levels of p52/p46ShcA, with GAPDH as loading control. (C) Increased expression of transfected p52ShcA in NMuMG cells decreased the TGF-β-induced Smad3 activation, assessed by anti-pSmad3 immunoblotting, and increased the TGF-β-induced Erk1/2 MAPK and Akt activation, assessed by immunoblotting for pErk1/2 and pAkt^S473^. Control panels show immunoblotting for Smad3, Erk1/2 MAPK, Akt, and GAPDH. (D) Increasing quantities of transfected p52ShcA plasmid, in μg per well, decrease the Smad3D407E association with TβRI, while increasing p52ShcA association, in TGF-β-treated 293T cells that were transfected to coexpress TβRII and TβRI. Smad3 or p52ShcA association with TβRI was visualized by anti-Flag immunoprecipitation of TβRI, followed by immunoblotting for Smad3 or HA-tagged p52ShcA, and Flag-tagged TβRI. Immunoblotting of the cell lysates shows p52/46ShcA levels, and TβRI and Smad3 levels, as well as GAPDH as loading control. (E) Increasing quantities of purified His-p52ShcA (shown in μg/incubation) interfere with binding of immunopurified Flag-tagged TβRI, isolated from TGF-β-treated 293T cells coexpressing TβRI and TβRII, to purified GST-Smad3. Immunoblotting of Flag-tagged TβRI adsorbed to glutathione-Sepharose-bound GST-Smad3 reveals association of Smad3 with TβRI in the absence of ShcA and loss of associated TβRI in the presence of ShcA, while anti-Smad3 immunoblotting reveals the GST-Smad3 used in the experiments. The lowest panel shows TβRI that was not bound to GST-Smad3. Comparing lane three with lane seven shows that dephosphorylation of TβRI using λ-phosphatase prevents association of Smad3 with TβRI. All experiments were reproducibly repeated at least three times. Supplemental data are shown in S6 Fig.

We have previously shown that increased TGF-β receptor levels at the cell surface confer increased autocrine TGF-β signaling [49–51]. Inhibition of ectodomain shedding, which enhances TβRI cell surface levels [49], or high glucose or insulin, which induce a rapid increase in TβRII and TβRI at the cell surface [50,51], both increase autocrine TGF-β signaling and TGF-β responsiveness. We therefore examined the cell surface TGF-β receptor levels using cell surface protein biotinylation in cells with decreased ShcA expression in comparison with control cells. Down-regulation of ShcA expression did not result in increased cell surface levels of TβRI or TβRII (Fig 6A; S6A and S6B Fig), nor did it enhance the TβRI and TβRII mRNA expression (S6C and S6D Fig). We conclude that the increased autocrine TGF-β signaling does not result from increased cell surface levels of TGF-β receptors. Additionally, down-regulation of ShcA expression did not enhance the expression or release of TGF-β1 (S6E and S6F Fig), the major TGF-β made by NMuMG cells in culture, nor did it affect the generation of active TGF-β (S6F Fig) that could otherwise have accounted for increased autocrine TGF-β signaling.

We next explored whether inhibition of TGF-β-induced Smad activation by ShcA might explain the increased Smad activation when ShcA expression is decreased. Indeed, increasing levels of transfected p52ShcA expression plasmid, and thus of p52ShcA expression, decreased the level of TGF-β-induced Smad3 activation (Fig 6B; S6G Fig). The decrease in Smad3 activation with increased p52ShcA expression correlated with enhanced levels of TGF-β-induced Erk MAPK and Akt activation (Fig 6C). To visualize whether ShcA affected the transient interaction of Smad3 with TβRI that is required for Smad3 activation, we used a mutant of Smad3 with Asp407 replaced by Glu, which has enhanced affinity for TβRI, thus allowing detection of the TGF-β-induced Smad3-TβRI interaction by immunoprecipitation [52]. Increasing ShcA expression resulted in a decreased interaction of Smad3D407E with the TGF-β-activated TβRI (Fig 6D; S6H Fig). Since ShcA can interact with TβRI [12], this result suggests that Smad3 and ShcA may compete for binding to TβRI. To address directly the nature of the competition, we purified GST-Smad3 and His-tagged p52ShcA from *Escherichia coli*. Immunopurified Flag-tagged TβRI, obtained from TGF-β-stimulated cells expressing tagged TβRII and TβRI, associated in vitro with glutathione-bound, purified GST-Smad3, and this interaction was prevented following dephosphorylation of TβRI by lambda phosphatase (Fig 6E; compare lanes three and seven). Purified His-tagged p52ShcA prevented the association of GST-Smad3 with TβRI (Fig 6E). These results suggest that ShcA binding to TβRI interferes with Smad3 binding to TβRI, and that steric incompatibility of ShcA and Smad3 binding may prevent TGF-β-induced Smad3 activation in the direct presence of ShcA. These results further suggest that ShcA expression is an important determinant of TGF-β-induced Smad activation.

### ShcA Regulates Subcellular Compartmentalization of TβRI

**Fig 7:**
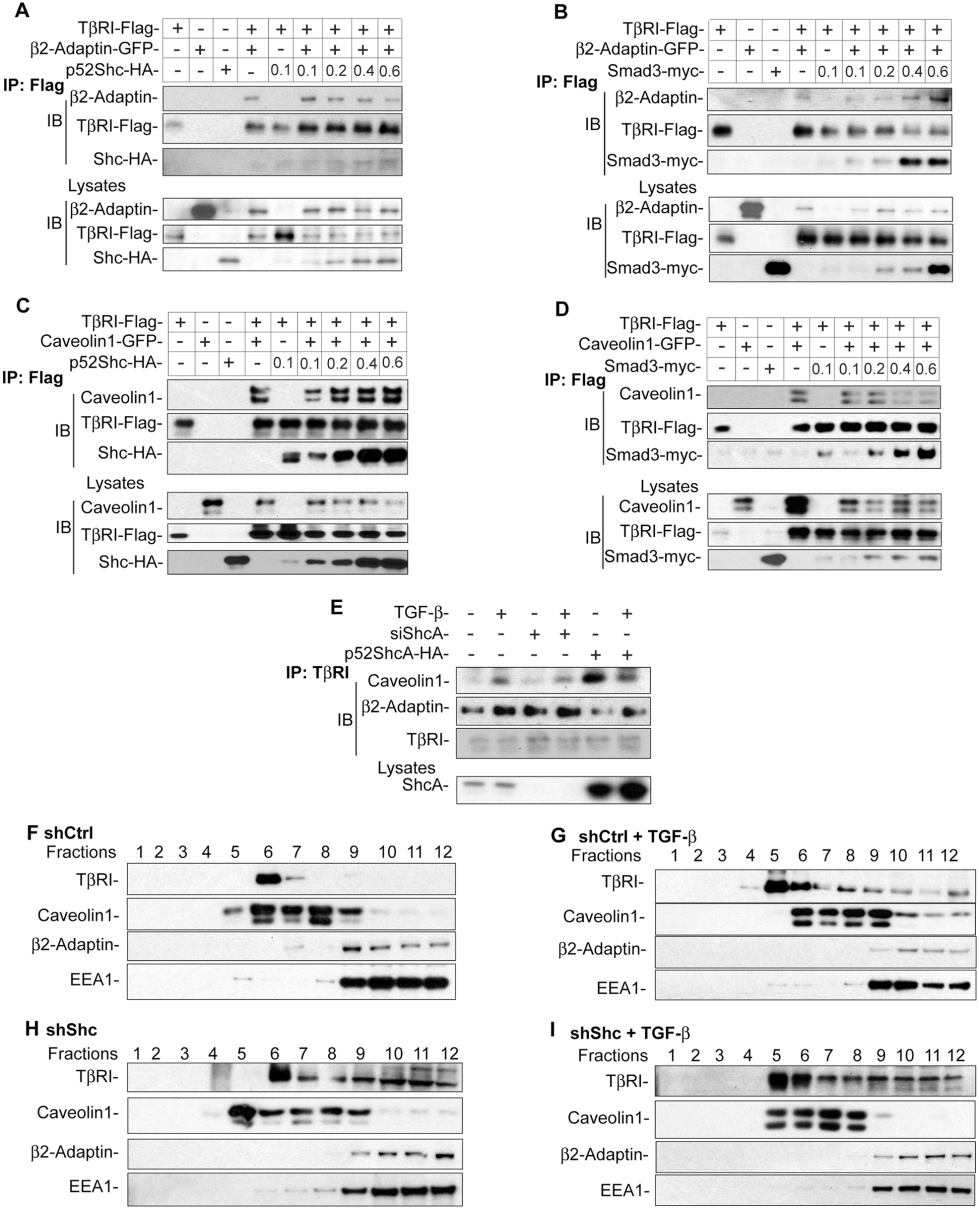
ShcA defines the compartmentalization of TβRI. (A–D) 293T cells, coexpressing TβRII and Flag-tagged TβRI, were transfected to additionally express GFP-tagged β2-adaptin (A, B) or caveolin 1 (C, D), with or without increasing levels of HA-tagged p52ShcA (A, C) or myc-tagged Smad3 (B, D), as shown with the amount of plasmid (μg) per well. Following stimulation of the cells with TGF-β, Flag-tagged TβRI was immunoprecipitated and associated β2-adaptin (A, B) or caveolin 1 (C, D) were visualized by immunoblotting. The lower panels show the expression of Flag-tagged TβRI, β2-adaptin (A, B), or caveolin-1 (C, D) in the cell lysates, visualized by immunoblotting. (E) NMuMG cells were transfected with siRNA to down-regulate ShcA expression or with p52ShcA expression plasmid to enhance its expression, and treated or not with TGF-β. Endogenous TβRI was immunoprecipitated, and associated β2-adaptin or caveolin 1 were visualized by immunoblotting. (F–I) Association of TβRI with clathrin-coated membranes and caveolar microdomains, evaluated following sucrose gradient centrifugation. NMuMG cells lentivirally infected to express control shRNA (F, G), or ShcA shRNA to down-regulate ShcA expression (H, I), were treated (G, I) or not (F, H) with TGF-β. Following removal of the nuclei, the cell lysates were fractionated by sucrose gradient centrifugation, and the fractions were immunoblotted for TβRI, caveolin-1, clathrin heavy chain, or the EEA1 early endosomal marker. All experiments were reproducibly repeated at least three times. Supplemental data are shown in S7 Fig.

Since p52ShcA interfered with the association of Smad3 with TβRI and promoted TGF-β-induced Erk MAPK activation, we evaluated whether it affected the compartmentalization of TβRI in clathrin-coated pits, where TGF-β-induced Smad signaling is initiated [53,54]. The TβRI receptor associates and coimmunoprecipitates with the β2-adaptin subunit of the AP2 adaptor complex [55], which mediates clathrin-dependent endocytosis from the plasma membrane [56]. Accordingly, in TGF-β-treated cells transfected to express both TβRII and Flag-tagged TβRI, TβRI coprecipitated with β2-adaptin, and p52ShcA decreased this association (Fig 7A; S7A Fig). Conversely, increasing levels of Smad3 enhanced the association of β2-adaptin with TβRI (Fig 7B; S7B Fig).

The TβRI receptor has also been shown to interact with caveolin 1 [57], a cholesterol-binding protein that is the major component of caveolae [58,59]. Increasing ShcA expression enhanced the association of TβRI with caveolin 1 in TGF-β-stimulated cells (Fig 7C; S7C Fig). In contrast, increasing the levels of coexpressed Smad3 decreased the interaction of TβRI with caveolin 1 (Fig 7D; S7D Fig). Finally, decreasing the endogenous level of ShcA expression using siRNA modestly decreased the TGF-β-induced association of endogenous TβRI and caveolin 1 in NMuMG cells while increasing the association of TβRI with β2-adaptin (Fig 7E; S7E and S7F Fig). Conversely, ShcA overexpression increased the interaction of TβRI with caveolin 1 and decreased its interaction with β2-adaptin (Fig 7E; S7E and S7F Fig). Together, these data support the notion that ShcA controls the partitioning of the TβRI receptors between the Smad-activating receptor complexes in clathrin-coated endosomes and caveolar microdomains.

A role of ShcA in defining this balance was further supported by sucrose gradient fractionation of lysates of NMuMG cells. In control cells, TβRI cofractionated with caveolin-1, with only minimal levels of TβRI in the fractions containing clathrin and the early endosomal marker, EEA1 (Fig 7F). TGF-β treatment induced association of some TβRI with the fractions containing clathrin and EEA1 (Fig 7G), as expected since TGF-β induces Smad activation. Decreasing ShcA expression following lentiviral expression of ShcA shRNA resulted, in the absence of TGF-β treatment, in a less confined association of TβRI with caveolin-1 and a broader association of TβRI with the clathrin/EEA1 fractions (Fig 7H). This increased TβRI association with clathrin/EEA1 fractions is consistent with ShcA’s role in sequestering TGF-β receptor complexes in caveolar microdomains, suggested by immunoprecipitation analyses (Fig 7A–7E). It is also consistent with our finding that reduced ShcA expression results in increased autocrine TGF-β-induced Smad activation, which occurs in clathrin/EEA1 endosomes. The TβRI association with clathrin/EEA1 fractions was not much enhanced upon adding TGF-β (Fig 7I).

## Discussion

The ability of TGF-β to induce EMT is explained in part by the Smad-directed activation of EMT master transcription factor expression and functional cooperation of Smads with these transcription factors; yet, synergy with non-Smad signaling is essential for the realization of EMT [24,26]. Since TGF-β induces Erk MAPK pathway activation through Tyr phosphorylation of p52ShcA, we addressed the role of ShcA in TGF-β-induced EMT of nontransformed epithelial cells. Unexpectedly, we found that the expression level of ShcA plays a role in the maintenance of the epithelial phenotype and in EMT, by balancing TGF-β-induced signaling. Specifically, our results revealed that (1) ShcA represses TGF-β-induced Smad activation, thus repressing autocrine TGF-β/Smad signaling, (2) ShcA enables differential compartmentalization of Smad signaling and ShcA-mediated non-Smad signaling in response to TGF-β, (3) ShcA expression helps to maintain the epithelial phenotype, and decreased ShcA expression promotes EMT resulting from increased autocrine TGF-β/Smad signaling.

### ShcA Controls TGF-β-Induced Smad Activation and Signaling Responses

We previously reported that p52ShcA and p66ShcA can associate with the TβRI receptor and are phosphorylated by its kinase on Ser and Tyr, and that TGF-β-induced recruitment and Tyr phosphorylation of p52ShcA by TβRI enables TGF-β-induced Erk MAPK activation [12]. To address the role of ShcA in TGF-β-induced EMT, we used two epithelial cell lines, NMuMG and HaCaT cells that are commonly used to study EMT. Like most cells in culture, they express predominantly the p52 isoform of ShcA with lower levels of p66ShcA. Down-regulation of ShcA expression repressed the expression of all ShcA isoforms; targeted down-regulation of only p52ShcA cannot be done with the p52ShcA sequence fully comprised in p66ShcA. Our results demonstrate that decreasing ShcA expression enhances Smad signaling and, thus, that ShcA acts to repress Smad activation. This was apparent in the level of Smad3 activation and nuclear import in response to TGF-β and the activation of TGF-β/Smad target gene expression. These findings are consistent with results on the control of TGF-β signaling by a mutant p53 in human prostate carcinoma cell lines, showing that Smad2/3 activation is decreased upon overexpression of ShcA and enhanced when ShcA levels are reduced [60]. The increased Smad3 activation when ShcA expression is down-regulated did not result from an increase in cell surface TGF-β receptors or from generation of active TGF-β by the cells, but from enhanced Smad3 recruitment to the activated TβRI. We found that p52ShcA attenuates TGF-β-induced association of Smad3 with TβRI through direct competition, as shown in cells coexpressing these signaling mediators and using purified proteins. As a result of this interference, ShcA expression, and in particular p52ShcA, acts as a cell-intrinsic determinant in the control of TGF-β-induced Smad activation and gene expression responses. p66ShcA has properties that are distinct from p52ShcA, e.g., through functional linkage with the oxidative stress response [61,62] and was shown to oppose p52ShcA in RTK-induced Erk MAPK activation [63]. Whether p66ShcA also controls TGF-β-induced Smad and Erk MAPK activation, e.g., through competition with p52ShcA, remains to be seen.

### ShcA Defines the Differential Compartmentalization of TGF-β-Induced Smad Signaling

**Fig 8:**
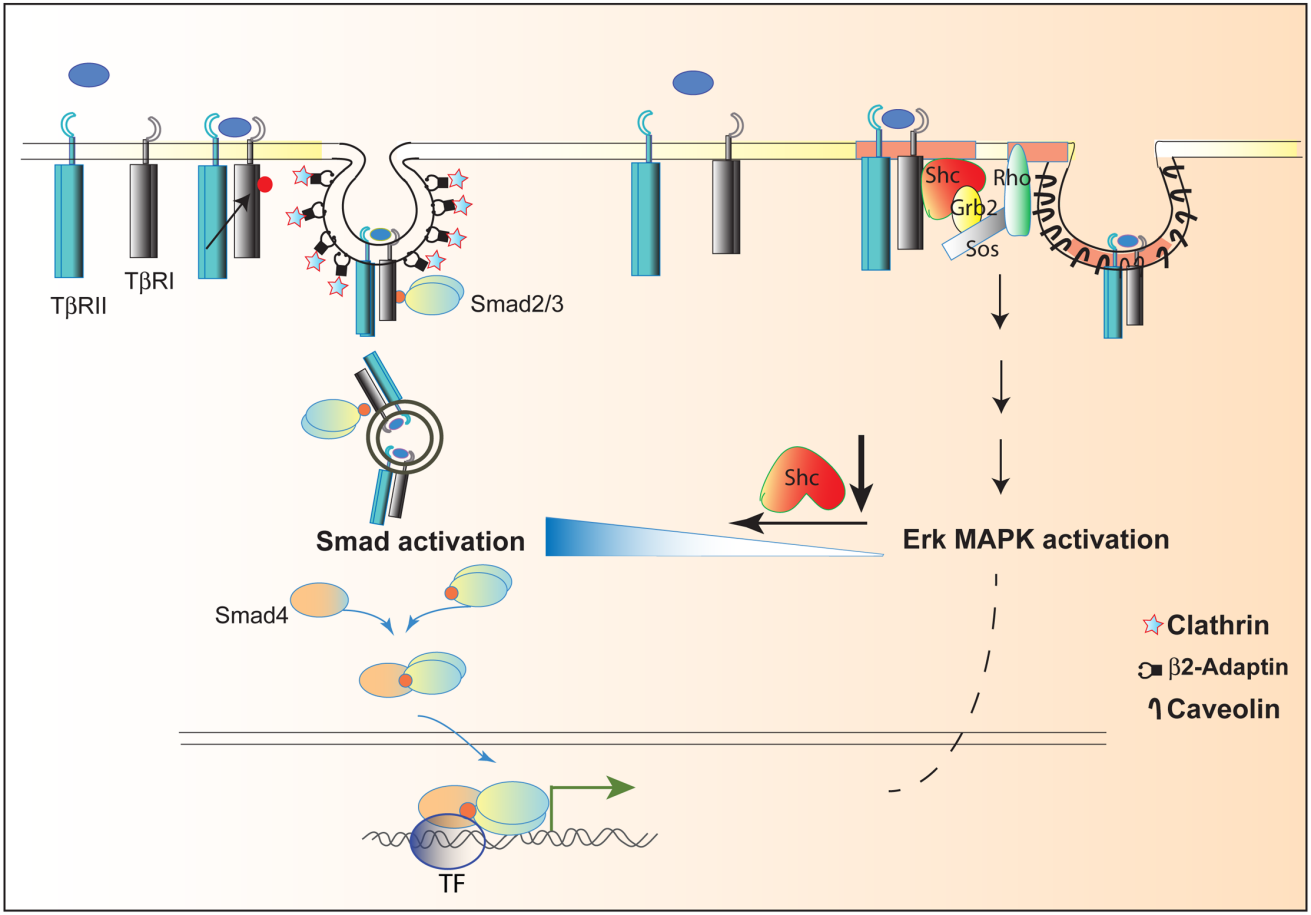
Schematic diagram showing the distribution of hetero-tetrameric TGF-β receptor complexes between clathrin-coated pits and caveolae. ShcA stabilizes the TGF-β receptor complexes in the caveolae, thus enabling TGF-β-induced Erk MAPK signaling. Decreasing ShcA expression results in increased levels of TGF-β receptor complexes in clathrin-coated pits, resulting in enhanced Smad signaling.

TGF-β-induced Smad activation occurs in clathrin-coated pits [53,54], and TGF-β receptors associate with β2-adaptin in clathrin-associated AP2 complexes [55]. Conversely, TGF-β receptors are enriched in lipid-rich caveolae [54,64], and the TβRI receptor can associate with caveolin 1 [57]. Whereas TGF-β receptor degradation in caveolar microdomains dampens TGF-β responsiveness [54], cholesterol depletion experiments correlate TβRI localization in cholesterol-rich lipid rafts with TGF-β-induced Erk MAPK signaling [64]. Additionally, TGF-β-induced Akt activation requires caveolin [65]. Our results now argue that ShcA controls TGF-β responsiveness by defining the distribution of TGF-β receptor complexes between clathrin-coated pits and cholesterol-rich caveolae (Fig 8).

We show that p52ShcA expression stabilizes the TβRI interaction with caveolin 1, while decreasing the TβRI association with the AP2 complex, consistent with the decreased TGF-β-induced Smad3 activation, and that depletion of ShcA expression decreases the TGF-β-induced TβRI interaction with caveolin 1, enabling a higher level of TβRI interaction with AP2. Membrane fractionation revealed that most TGF-β receptor complexes colocalize with caveolin 1 microdomains in unstimulated cells, and that ShcA down-regulation shifted a fraction of the receptors into clathrin-containing endosomal membranes, enabling increased Smad2/3 activation in response to autocrine TGF-β. We therefore conclude that ShcA controls the compartmentalized distribution of TGF-β receptors between clathrin-coated pits and caveolae (Fig 8). Consistent with our observation that p52ShcA links TGF-β receptor activation with TGF-β-induced Erk MAPK activation [12], ShcA appears to balance the TGF-β response through Smad versus Erk MAPK pathway signaling. In RTK signaling or signaling through receptor-associated Tyr kinases, p52ShcA phosphorylation in caveolar membranes facilitates Erk MAPK activation [58,66,67]. The dual specificity kinase TGF-β receptors allow for a similar mode of Erk MAPK pathway activation in response to TGF-β, albeit to a much lower extent. Our findings also complement a recent observation that Dab2, an SH2 domain adaptor that interacts with TβRI and clathrin, and is required for TGF-β-induced Smad signaling, helps control the TβRI localization in clathrin endosomes and enhances its clathrin-mediated endocytosis [68]. It is conceivable that p52ShcA and Dab2 may have opposing roles in balancing Smad activation versus non-Smad responses.

Previous studies revealed that cells regulate their TGF-β responsiveness by controlling the levels of functional TGF-β receptors at the cell surface. High glucose and insulin enhance TGF-β responsiveness by inducing a rapid mobilization of TβRII and TβRI from intracellular stores to the cell surface [50,51]. Conversely, cleavage of cell surface TβRI by the membrane-associated metalloprotease TACE decreases the cell sensitivity to TGF-β [49]. Our findings now present an alternative mechanism of controlling the TGF-β responses, in which ShcA defines the compartmentalization of cell surface TGF-β receptor complexes, balancing Smad-mediated responses against Erk MAPK pathway activation through ShcA. Compartmentalized balancing of TGF-β receptor signaling by ShcA may provide an as-yet-unappreciated level of control with scenarios that impact cell homeostasis and cancer progression, as increasingly valued for the spatial (de)regulation of RTK signaling [69].

### Decreased ShcA Expression Attenuates the Epithelial Integrity and Promotes EMT

While much attention is given to the roles of Smads in driving the EMT gene expression program [24], the MEK1/2-Erk MAPK pathway was shown to be required for TGF-β-induced EMT [35,70], and blocking Akt or mTOR complex 2 activation prevents transition of epithelial cells into the mesenchymal phenotype [34,71]. These observations support a model that the Erk MAPK and Akt-mTOR pathways cooperate with Smad signaling in the elaboration of EMT [24]. In the epithelial cells studied, decreasing ShcA expression resulted in attenuated Erk MAPK activation, consistent with the observation that targeted inactivation of ShcA expression attenuates growth factor-induced Erk MAPK activation [6], and with the presence of growth factors in serum that act through RTKs and thus activate Erk MAPK signaling. Down-regulated ShcA expression also attenuated Akt activation, suggesting a role of ShcA in coupling growth factor signaling to Akt activation, as proposed for some RTKs [2–4]. That increased Smad signaling in the context of attenuated Akt and Erk MAPK pathway activation leads to EMT in response to autocrine TGF-β signaling highlights the role of TGF-β-induced Smad signaling in EMT but does not argue against a requirement for Erk MAPK and Akt.

Our finding in nontransformed cells that ShcA protects epithelial cells from transitioning toward a mesenchymal phenotype, by repressing TGF-β/Smad activation, raises the possibility that carcinoma cells control through ShcA the EMT phenotype and, consequently, the invasive and stem cell phenotype and cancer dissemination. ShcA is expressed in many cell types [2], but little is known about the control of ShcA expression, and most studies do not distinguish p52ShcA from p66ShcA expression, even though p66ShcA and p52ShcA have distinct functions [1,2,72]. Consistent with its role in mitogenic signaling [2], ShcA is required for breast cancer development in mice [73]. Immunohistochemistry, however, reveals heterogeneity in ShcA expression among carcinomas with levels that are often lower than those in normal epithelia [74]. Moreover, mammary carcinoma cells that express a mutant ShcA lacking a functional phosphoTyr-binding PTB domain show increased expression of mesenchymal fibronectin and α5β1 integrin [75], arguing that with impaired ShcA function the carcinoma cells might transition toward a mesenchymal phenotype. Distinct functions of p66SchA, with some antagonizing those of p52SchA [1,2,60] may explain the complex roles of SchA in controlling epithelial plasticity of carcinomas. Accordingly, analyses of breast cancer cell lines suggest differential regulation of p52ShcA and p66ShcA expression [76]. It is tempting to speculate that carcinoma cells may down-regulate ShcA, or specifically p52ShcA expression, at sites of invasion, where cells undergo EMT. Whether differential p52ShcA and p66ShcA expression correlates with carcinoma cell behavior is an open question. Although in our nontransformed epithelial cells the ShcA expression level defines the sensitivity to EMT through modulation of TGF-β signaling, crosstalk of ShcA with oncogenic signaling may confer a more complex role of ShcA in the epithelial plasticity of cancer cells. Indeed, ShcA cooperates with Neu/ErbB2 signaling in the control of cell motility and invasion in transformed epithelial cells [77], through effects on focal adhesion turnover [5,77], and increased p52/46ShcA levels enhance migration of prostate carcinoma cells [60]. Additionally, p66ShcA overexpression promotes EMT in ErbB2-driven breast cancer cells, through up-regulated activation of the c-Met receptor by its ligand hepatocyte growth factor (HGF) [76]. It is unknown whether in this context p66ShcA antagonizes the role of p52ShcA, as seen in RTK responses [2,63,72,78]. Furthermore, it remains to be seen whether ShcA contributes to epithelial plasticity responses when EMT is driven by increased RTK or Wnt signaling. Finally, consistent with the linkage of EMT with stem cell generation [31,32,41], decreased ShcA expression promoted stem cell generation, apparent by marker expression and mammosphere formation. Future studies will reveal whether p52ShcA and p66ShcA expression control cancer stem cell generation and tumor initiation.

## Materials and Methods

### Cell Culture Reagents and Antibodies

NMuMG, HaCaT, and 293T cells were cultured in DMEM with 10% FBS. NMuMG culture medium was supplemented with 10 μg/ml insulin (Sigma) for maintenance. Cells were treated with 2 ng/ml TGF-β1 (HumanZyme), 5 μM SB431542 (Sigma) or 5 μM LY2109761 (SelleckChem) for the indicated times. U0126 (Calbiochem), SB203580 (Calbiochem) and LY294002 (Sigma-Aldrich) were used at 7 μM, 20 μM, and 2.5 μM, respectively. The neutralizing panTGF-β inhibitor monoclonal antibody [79] was used at 200 ng/ml. For immunoprecipitations and/or immunoblotting, we used antibodies to ShcA, EEA1, caveolin-1, β2-adaptin, and clathrin heavy chain from BD Biosciences, GAPDH, CD49f, and CD24 (from Santa Cruz Biotechnology), TβRI and TβRII (Abcam and Santa Cruz Biotechnology), phosphoAkt (Ser473), and Akt, phosphoSmad3, Smad3, phosphoErk, Erk, phospho-p38, p38, E-cadherin, N-cadherin, vimentin, and fibronectin from Cell Signaling. Anti-Flag M2 (Sigma), anti-HA.11 (Covance), anti-GFP (Rockland and Aves labs), and anti-Myc 9E10 (Covance) were used for immunoprecipitation of tagged proteins.

### Plasmids, Cell Culture, and Transfection

The expression plasmids for C-terminally Flag-tagged TβRI or Myc-tagged TβRII [80], C-terminally haemagglutinin (HA)-tagged p52ShcA [12] and C-terminal Myc-Smad3D407E [52] were described. An expression plasmid encoding His-tagged p52ShcA [81] was a gift from Dr. John Ladbury. The expression plasmids for GFP-tagged β2 adaptin [55] and GFP-tagged caveolin-1 [82] were provided by Dr. Ed Leof (Mayo Clinic) and Dr. Martin A. Schwartz (Yale School of Medicine), respectively. Control siRNA and siRNA oligonucleotides targeting mouse or human ShcA [83] were from Qiagen (Table 1). Lentiviral vectors expressing control shRNA or shRNA targeting human and mouse ShcA were from Sigma-Aldrich (Table 2). For plasmid transfections, NMuMG, HaCaT, or 293T cells were plated in six-well plates and transfected with Lipofectamine 2000 (Invitrogen) or Xtreme HP (Roche). Five hours after transfection, cells were transferred to fresh medium-containing 10% FBS and incubated for 24–48 h. For siRNA transfections, NMuMG or HaCaT cells were plated in six-well plates and transfected with RNAiMax (Invitrogen). Eight to twelve hours after transfection, cells were transferred to fresh medium containing 10% FBS, cultured for another 12 h, followed by a second siRNA transfection and incubation for an additional 48–72 h.

**Table 1:** shRNA sequences.

shRNAs	**ShcA-Human**
3’UTR	CCGGGCCTATGTACTCTACGCCAAACTCGAGTTTGGCGTAGAGTACATAGGCTTTTTG
CDS	CCGGCCACATGCAATCTATCTATCTCATTCTCGAGAATGAGATAGATTGCATGTGGTTTTTG
CDS	CCGGCCACGGGAGCTTTGTCAATAACTCGAGTTATTGACAAAGCTCCCGTGGTTTTTG
	**ShcA-Mouse**
CDS	CCGGGCCGACTGCAAACAGATCATTCTCGAGAATGATCTGTTTGCAGTCGGCTTTTTG
CDS	CCGGGCCATCAGTTTGGTGTGTGAACTCGAGTTCACACACCAAACTGATGGCTTTTTG
CDS	CCGGGCTGAGTATGTTGCCTATGTTCTCGAGAACATAGGCAACATACTCAGCTTTTTG

**Table 2:** siRNA sequences.

siRNAs	**ShcA-Mouse**
siShc-a	5’-CTGAAGTTTGCTGGAATGCCA-3’
siShc-b	5’-ACACGGGAGCTTTGTCAATAA-3’
	**ShcA-Human**
siShc-c	5’-CTGAAATTTGCTGGAATGCCA-3’
siShc-d	5’-CCACGGGAGCTTTGTCAATAA-3’

### Stable Cell Lines

NMuMG or HaCaT cells were infected with lentiviral vectors expressing shRNA against mouse or human ShcA or TβRI (Sigma-Aldrich). The lentiviral vector pLKO.1 was used to generate control cells (Sigma-Aldrich). Following infection, the cells were selected with 1 μg/ml or puromycin (InvivoGen) for a week. Stably infected cell populations were generated as described [49]. The expression levels of ShcA or TβRI were assessed by immunoblotting with anti-ShcA or anti-TβRI antibody. Target sequences of shRNAs to silence the expression of human or mouse ShcA are shown in Table 1.

### Three-Dimensional (3-D) Cell Cultures

NMuMG cells, infected with lentiviral vector expressing control or ShcA shRNA, were grown to confluence, washed twice with PBS and trypsinized. Cells were seeded in eight chamber culture slides (BD Biosciences) coated with growth factor-reduced BD Matrigel matrix (BD Biosciences) at a density of 6,250 cells/ml/well in DMEM with 2% Matrigel in the absence or presence of 2 ng/ml TGF-β. Cultures were supplemented with fresh medium every 2 or 3 d. Cell morphology was observed after seven days using a phase-contrast microscope (DMI5000, Leica Microsystems), and pictures were acquired and analyzed using Photoshop CS5 software.

### Microscopy and Immunofluorescence

Cells plated on chamber slides were fixed with 4% paraformaldehyde (PFA) for 20 min, permeabilized with PBS containing 2% PFA and 0.2% Triton X-100 (PBT) for 15 min and blocked with 2.5% BSA for 1 h. The slides were incubated with antibodies to E-cadherin (BD Biosciences), fibronectin (BD Biosciences), or Smad2/3 (BD Biosciences) at a 1:200 to 1:500 dilution at 4°C overnight, and then stained for 2 h with secondary antibodies conjugated to Alexa Fluor-488 or -647 (1:500 dilution, Invitrogen) at room temperature. Phalloidin (Life Technologies) was used at a 1:500 dilution along with secondary antibodies to stain actin filaments. The slides were mounted with Prolong Gold antifade reagent (Invitrogen) with DAPI to visualize nuclei. The cells were viewed with an inverted light microscope (DMI5000, Leica Microsystems) or a laser scanning confocal microscope (SP5, Leica Microsystems). Cell morphology was evaluated using a phase-contrast microscope (DMI5000, Leica Microsystems). Images were analyzed using Leica application suite (Leica Microsystems), Axiovision (Carl Zeiss MicroImaging, Inc.), ImageJ, and/or Adobe Photoshop CS5.

### Rescue of ShcA Expression

To stably down-regulate ShcA expression, HaCaT cells were infected with lentivirus expressing shRNA targeting the 3’UTR of ShcA or the empty lentiviral vector pLKO.1, and selected with 3 μg/ml puromycin for one week. Control cells or cells with decreased ShcA expression were then transfected with 0.2 μg of p52ShcA plasmid using Lipofectamine 2000 (Invitrogen). p52ShcA expression was evaluated by immunoblot at 24 h after transfection, and the cell morphology was monitored by phase contrast microscopy after 36 h. Immunofluorecence for E-cadherin and fibronectin and quantification of E-cadherin and fibronectin mRNA by qRT-PCR were also done at that time.

### Migration Assays

Confluent cell monolayers in DMEM with 10% FBS were wounded with a 10 μl plastic tip, and migration assays were performed as described [33], using a Leica DMI 4000B microscope and a Leica DFC 350FX camera, with photographs taken at 0 h and 14 h.

### Invasion Assays

Invasion assays, performed as described [33], utilized cells treated with or without 2 ng/ml TGF-β for 36 h, and 50,000 cells added to Matrigel-coated inserts (BioCoat Matrigel Invasion Chamber; Becton Dickinson) in DMEM, 0.2% FBS. These were then placed in companion plates with DMEM 10% FBS for 24 h. After removal of the cells in the upper chambers, the filters were fixed in methanol for 5 min at –20°C, and mounted using Prolong Gold Antifade reagent with DAPI (Invitrogen). The DAPI-stained cells that transversed the filter were counted using DMI 5000 Leica microscope.

### Cell Dissemination in Zebrafish Embryos

Adult zebrafish were maintained in a zebrafish facility with a 14:10 day:night cycle and handled in compliance with an approved institutional protocol. Control NMuMG cells and cells with down-regulated ShcA expression were grown to confluence, washed twice with PBS, trypsinized and labeled with CM-DiI by immersion for 5 min at 37°C, and then transferred to ice for 15 min, as instructed by the manufacturer. Cells were then washed three times with PBS, suspended in PBS, and transferred into a borosilicate needle for injection into anesthetized dechorionated embryos, held on agarose-lined plates. 100–150 DiI-labeled cells were injected in the mid yolk sac region of the zebrafish embryos. After injection, embryos were sorted for fluorescence, and pictures were taken. Subsequently, the xenografted embryos were held at 31–32°C for 3 d prior to imaging of cell dissemination. Larvae were anesthetized with 0.003% tricaine (Sigma), and pictures were taken using a Leica M205 microscope. For each condition, the data shown are from at least two experiments with at least fifty embryos per group. Images were analyzed and modified for brightness and contrast using Adobe Photoshop CS5 software.

### Mammosphere Formation Assay

Cells were seeded on ultra-low attachment plates [42] at a density of 200 cells/200 μl/well in MEGM medium (Lonza) supplemented with B27, 10 ng/ml bFGF, 20 ng/ml EGF. After incubating the cells for 4–5 d, the primary mammospheres were counted and visualized using a DMI 5000 Leica microscope. For secondary mammosphere formation assays, mammospheres were collected by centrifugation at 800 g for 5 min, resuspended in 100 μl of 0.05% trypsin and incubated at 37°C for 10 min, and further dissociated into single cells by pipetting, as verified by microscopy. Single cell dilutions were then replated on ultralow attachment plates, and colonies were quantified as for the primary mammosphere assays.

### Immunoblotting and Immunoprecipitation

Cells were lysed in lysis buffer (25 mM Tris-HCl pH 7.5, 150 mM NaCl, 2 mM EDTA, 0.1% EDTA, 0.7% Triton X-100, 10% glycerol) and protease inhibitor cocktail (Roche or ThermoScientific). For immunoblotting, proteins were quantified using Bio-Rad protein assay (Bio-Rad Laboratories), and 20–80 μg of protein was separated by SDS-PAGE and transferred to 0.45 μm PVDF membrane. Membranes were blocked in TBS, 0.1% Tween 20, and 5% BSA or 5% milk in TBS for 1 h to 6 h, followed by overnight incubation with primary antibody diluted at 1:500–1:5,000 in blocking solution and 1–2 h incubation with HRP-conjugated secondary antibodies diluted at 1:5,000–1:20,000. Immunoreactive protein was detected using ECL (GE Healthcare and Perkin Elmer) and BioMax film (Kodak and Denville). For immunoprecipitation, NMuMG, HaCaT, and 293T cells were harvested at 24 or 48 h after transfection and lysed in lysis buffer. Lysates were subjected to immunoprecipitation with anti-Flag M2, anti-HA, or anti-Myc antibody and protein G-Sepharose 4 fast flow (GE Healthcare). Immune complexes were washed three times with lysis buffer and subjected to immunoblotting with anti-Flag, anti-Myc, or anti-HA antibodies.

For immunoprecipitation of proteins at endogenous levels, NMuMG or HaCaT cells were grown to 80% confluence in 100-mm cell culture dishes, serum starved for 4–12 h, treated with 2 ng/ml TGF-β or an inhibitor, washed with cold PBS, and lysed in lysis buffer. The lysates were precleared with rabbit or mouse IgG (Jackson ImmunoResearch Laboratory) and protein A/G Sepharose (GE healthcare), followed by immunoprecipitation with anti-TβR1 (Abcam) or ShcA (BD Biosciences) antibodies, control GFP antibody (Sigma-Aldrich), or IgG from the same species. Immune complexes were precipitated with protein A/G Sepharose (GE healthcare), and separated on SDS-PAGE followed by immunoblotting.

### Luciferase Assays

NMuMG and HaCaT cells were cultured in 12-well plates and transfected with SBE-binding firefly luciferase reporter plasmid [84], and a Renilla luciferase reporter under the control of the thymidine kinase promoter (Promega) was cotransfected as control. After 24 h transfection, cells were treated with or without 800 pg/ml of TGF-β. The luciferase activities of firefly and renilla were quantified after 6 h of 800 pg/ml TGF-β treatment using the Dual Luciferase Kit (Promega). The firefly luciferase activities were normalized to Renilla luciferase activity.

### Isolation of Nuclear and Cytoplasmic Extracts

Confluent NMuMG cells in 10 cm dishes were washed with ice-cold PBS and then harvested by scraping in hypotonic buffer (10 mM Hepes, 1.5 mM MgCl_2_, 10 mM KCl, 1 mM PMSF, and 0.5 mM DTT with Roche protease inhibitor Mini Complete). The cells were lysed using a prechilled Dounce homogenizer (10–15 strokes with a tight pestle). Cell lysates were subjected to centrifugation at 228 g for 5 min at 4°C to pellet nuclei, and the cytoplasmic supernatant fraction was collected. The nuclear pellet was then resuspended in RIPA buffer (25 mM Tris-HCl pH 7.5, 150 mM NaCl, 2 mM EDTA, 0.1% EDTA, 1.0% Triton X-100, 0.1% SDS, and 0.25% sodium deoxycholate) and sonicated with short sonication burst cycles of six times for 10 s. Protein concentrations in the nuclear and cytoplasmic extracts were determined using the Bradford assay.

### Protein Purification and Adsorption Assays

GST-fused Smad3 was generated in *E*. *coli* transformed with pGEX-Smad3 [85] and purified with Glutathione Sepharose 4B (GE Healthcare). 293T cells were transfected to express Flag-tagged TβRI and myc-tagged TβRII and treated with 2 ng/ml TGF-β for 30 min. The TβRI was immunopurified using anti-Flag M2 affinity agarose. His-tagged p52ShcA was purified as described.

Purified Flag-tagged TβRI was incubated for 20 min with mild shaking at 4°C with GST- Smad3 that was immobilized on glutathione-Sepharose. After three washes, the beads were incubated with His-tagged ShcA for 30 min at 4°C. After adsorption, bound and unbound proteins were separated by SDS-PAGE and analyzed by immunoblotting, as described [52].

### Detection of Cell Surface TGF-β Receptors

Cell surface TGF-β receptors were visualized by cell surface protein biotinylation as described [49]. Briefly, NMuMG cells were grown to confluence, serum starved for 4–6 h, treated with 2 ng/ml TGF-β for 30 min to 1 h, and labeled with Sulpho-NHS-LC Biotin (Thermo Scientific) at 4°C for 30 min. Cells were washed with 100 mM glycine and lysed in lysis buffer. Biotinylated cell surface proteins were adsorbed to neutravidin agarose (Thermo Scientific) and analyzed by immunoblotting with antibodies to TβRI or TβRII.

### TGF-β Activity Assay

Active TGF-β was quantified using TMLC reporter cells with an integrated TGF-β/Smad3-responsive luciferease expression unit [86]. NMuMG cells, transfected with control or ShcA siRNA, were grown in serum-free medium for 2 h, conditioned media were collected, concentrated using Pierce protein concentrator (Thermo scientific), and either kept at 4°C or heated at 80°C for 10 min to activate all latent TGF-β. The conditioned media samples were incubated with TMLC reporter cells in 12-well plates at a density of 150,000 cells/well for 12–16 h. In parallel, media samples with known concentrations of TGF-β were added to TMLC reporter cells to generate a standard curve. The luciferase activities of the conditioned media were calibrated against the standard curve TMLC reporter cells, allowing us to define the TGF-β concentration in conditioned media samples.

### Subcellular Fractionation

NMuMG and HaCaT cells were grown to 80% confluence, serum starved for 2 h, treated with 2 ng/ml TGF-β for 30 min to 1 h, washed with cold PBS, and lysed with 0.5 M sodium carbonate buffer pH 11.0. The subcellular fractionation was performed as described [54]. The cells were then homogenized using Dounce homogenizer with 15 tight strokes followed by three 20-s bursts of sonication (Vibra-Cell). The lysates were centrifuged for 800 g to remove debris, adjusted to 4 ml of 40% sucrose in 10 mM HEPES buffer, pH 7.5, and placed at the bottom of ultracentrifuge tubes. Then 30% sucrose was overlaid, followed by 5% sucrose, to generate by centrifugation a discontinuous 5%–40% sucrose gradient. Centrifugation was at 38,000 rpm for 16–18 h using a Beckmann Coulter Optima L-90K ultracentrifuge and rotor SW41. Twelve 1 ml fractions were collected from the top of the tube and analyzed by SDS-PAGE.

### qRT-PCR Analysis

To quantify ShcA, TβRI, E-cadherin, N-cadherin, fibronectin, Snail, Slug, Smad7, and PAI-I mRNA expression, NMuMG and HaCaT cells were treated with or without 2 ng/ml TGF-β, and RNA was isolated with RNeasy kit (Qiagen) and used as a template for reverse transcriptase. mRNAs were quantified by qRT-PCR with IQ SYBR green Supermix (Bio-Rad) and normalized against RPL19 mRNA. The primer sequences are shown in Table 3.

**Table 3:** qRT-PCR primer sequences.

Primers-Mouse	Forward (5’-----3’)	Reverse (5’-----3’)
RPL19	ATGAGTATGCTCAGGCTACAGA	GCATTGGCGATTTCATT GGTC-
Smad7	TCTGGACAGTCTGCAGTTGG	TCCTGCTGTGCAAAGTGTTC
PAI-I	TCTGGGAAAGGGTTCACTTTACC	GACACGCCATAGGGAGAGAAG
Snail	AAGATGCACATCCGAAGC	ATCTCTTCACATCCGAGTGG
Twist	CGGGTCATGGCTAACGTG	CAGCTTGCCATCTTGGAGTC
ZEB1	GCTGGCAAGACAACGTGAAAG	GCCTCAGGATAAATGACGGC
E-Cadherin	CAGGTCTCCTCATGGCTTTGC	CTTCCGAAAAGAAGGCTGTCC
Fibronectin	GCAGTGACCACCATTCCTG	GGTAGCCAGTGAGCTGAACAC
N-Cadherin	AGCGCAGTCTTACCGAAGG	TCGCTGCTTTCATACTGAACTTT
Vimentin	CGGCTGCGAGAGAAATTGC	CCACTTTTCCGTTCAAGGTCAAG
CD49f	TGCAGAGGGCGAACAGAAC	GCACACGTCACCACTTTGC
CD24	CCCACGCAGATTTATTCCAG	GACTTCCAGACGCCATTTG
**Human**		
Smad7	TGCTGTGAATCTTACGGGAAG	AATCCATCGGGTATCTGGAG
PAI-I	ATTCAAGCAGCTATGGGATTC	CTGGACGAAGATCGCGTCTG
Slug	TTTCTGGGCTGGCCAAACATAAGC	ACACAAGGTAATGTGTGGGTCCGA
Twist	GGAGTCCGCAGTCTTACGAG	TCTGGAGGACCTGGTAGAGG
ZEB1	GCACCTGAAGAGGACCAGAG	TGCATCTGGTGTTCCATTTT
E-Cadherin	TGCCCAGAAAATGAAAAAGG	GTGTATGTGGCAATGCGTTC
Fibronectin	TCCCTCGGAACATCAGAAAC	CAGTGGGAGACCTCGAGAAG
N-Cadherin	ACAGTGGCCACCTACAAAGG	CCGAGATGGGGTTGATAATG
Vimentin	GACGCCATCAACACCGAGTT	CTTTGTCGTTGGTTAGCTGGT

## Supporting Information

S1 Data Raw data for analyses shown in the Figures and Supplemental Figures, as indicated. Data include *p*-values for the statistical significance.
(XLSX)

S1 Fig Corresponding to Fig 1. Decreased ShcA expression promotes EMT in NMuMG and HaCaT cells. (A) Selective silencing of ShcA expression in NMuMG and HaCaT cells using transfected siRNA, i.e., siShc-a and siShc-b for NMuMG cells, and siShc-c and siShc-d for HaCaT cells. NMuMG and HaCaT cells were transfected with control or ShcA siRNA, and the immunoblotted p52ShcA band was quantified by densitometry. The graphs show averaged values of three independent experiments, relative to p52ShcA of control siRNA cells. Error bars indicate standard errors, based on three independent experiments. (B) ShcA mRNA quantified by qRT-PCR and normalized against RPL19 mRNA in NMuMG cells or HaCaT cells transfected with control siRNA or ShcA siRNA, i.e., siShc-a and siShc-b for NMuMG cells, or siShc-c and siShc-d for HaCaT cells. Error bars indicate standard errors, based on three independent experiments. (C, D) Immunoblots of EMT marker expression, i.e., E-cadherin, fibronectin, N-cadherin, and vimentin, in NMuMG (C) and HaCaT (D) cells were quantified by densitometry. The graphs show averaged values of three independent experiments, relative to the marker expression in control siRNA cells. Error bars indicate standard errors, based on three independent experiments. (E) Compared to cells expressing a control shRNA, HaCaT cells infected to express shRNA targeting the 3’UTR of ShcA mRNA showed decreased ShcA expression, assessed by immunoblotting. Subsequent transfection with an siRNA-insensitive expression plasmid encoding p52ShcA resulted in increased p52ShcA expression. GAPDH immunoblotting was used as loading control. (F) Phase contrast microscopic images of HaCaT cells transfected with control or shRNA against the 3’UTR of ShcA mRNA and then rescued or not with a transfected p52ShcA plasmid. (G) Immunofluorecence detection of E-cadherin and fibronectin expression in HaCaT cells generated in (E) and shown in (F). (H) Expression of E-cadherin and fibronectin mRNAs in HaCaT cells generated in (E), quantified by qRT-PCR and normalized to RPL19 mRNA. Error bars indicate standard errors, based on three independent experiments. All experiments were reproducibly repeated at least three times. *, *p* < 0.05. Supplemental data are shown in S1 Data.
(TIF)

S2 Fig Corresponding to Fig 2. Densitometry analyses of (A) p52ShcA immunoblots of the NMuMG cells that were used for the zebrafish injection assays (Fig 2C–2E), and (B) CD24 and CD49f expression in the NMuMG cells that were used for mammosphere analyses (Fig 2F and 2G). Error bars indicate standard errors, based on three independent experiments.
(TIF)

S3 Fig Corresponding to Fig 3. Effects of MEK1/2, PI3K, and p38 MAPK inhibition on the EMT phenotype of HaCaT cells with or without down-regulated ShcA expression. HaCaT cells transfected with control siRNA or ShcA siRNA (siShc-c) were treated or not with the MEK1/2 inhibitor U0126, the PI3K inhibitor LY294002, or the p38 MAPK inhibitor SB203580 for 36 h. In (A), the cell morphology was assessed by phase contrast microscopy, whereas in (B) Slug mRNA was quantified by qRT-PCR and normalized to RPL19 mRNA. The graphs show averaged values of three independent experiments, with error bars indicating standard errors, based on three experiments. Statistical analyses were performed using two-tailed two-sample unequal variance *t* test. *, *p* < 0.05. Supplemental data are shown in S1 Data.
(TIF)

S4 Fig Corresponding to Fig 4. The TβRI kinase activity is required for EMT in epithelial cells with down-regulated ShcA expression. (A) Decreasing ShcA expression, upon transfection of two different siRNAs targeting ShcA, but not control siRNA, enhances Snail mRNA expression in NMuMG cells, in the absence of or in response to 2 ng/ml TGF-β for 6 h, and SB431542 prevents the enhanced Snail mRNA expression. mRNA levels were quantified by qRT-PCR and normalized to RPL19 mRNA. Error bars indicate standard errors, based on three independent experiments. (B, C) LY2109761 and TGF-β monoclonal antibody inhibit the increase in Snail mRNA (B) or fibronectin mRNA (C) in cells transfected with two different siRNAs targeting ShcA. (D) Effects of SB431542, LY2109761 or pan–anti-TGF-β monoclonal antibody on the expression of E-cadherin or fibronectin and actin organization in NMuMG cells transfected with control siRNA or ShcA siRNA (siShc-a), assessed by immunofluorescence. DAPI staining visualized the nuclei. (E) Effects of SB431542 on the expression of E-cadherin, fibronectin, and vimentin in NMuMG cells, transfected with control siRNA or ShcA siRNA (siShc-a), assessed by immunoblotting. GAPDH immunoblotting provided the loading control. (F, G) Effects of SB431542 on the expression of vimentin (F) and N-cadherin (G), and actin organization (F, G) in NMuMG cells transfected with control siRNA or ShcA siRNA (siShc-a), assessed by immunofluorescence. DAPI staining visualized the nuclei. (H) Decreasing ShcA expression, upon transfection of two different siRNAs targeting ShcA, but not control siRNA, enhances Slug mRNA expression in HaCaT cells, in the absence of or in response to 2 ng/ml TGF-β for 6 h, and SB431542 prevents the enhanced Slug mRNA expression. mRNA levels were quantified by qRT-PCR and normalized to RPL19 mRNA. Error bars indicate standard errors, based on three independent experiments. *, *p* < 0.05. All experiments were reproducibly repeated at least three times. Supplemental data are shown in S1 Data.
(TIF)

S5 Fig Corresponding to Fig 5. (A, B) Immunoblots of Smad3 in nuclear and cytoplasmic fractions of NMuMG cells transfected with ShcA siRNA (siShc-a) or control siRNA. Histone H3 and GAPDH serve as nuclear and cytoplasmic controls, respectively. Densitometric analyses of three independent experiments with standard errors are shown in B. (C, D) Decreasing ShcA expression, upon transfection of two different siRNAs targeting ShcA, but not control siRNA, enhances Smad3-mediated transcription, quantified by luciferase expression from a 4xSBE-luciferase reporter and normalized against the cotransfected Renilla-Lux reporter, in NMuMG (C) and HaCaT (D) cells, in the absence of or in response to 0.8 ng/ml TGF-β, or treated with SB431542 for 6 h. The TβRI kinase inhibitor SB431542 inhibits the luciferase expression. (E–H) Decreasing ShcA expression, upon transfection of NMuMG (E, G) and HaCaT (F, H) cells with ShcA siRNA (siShc-a in NMuMG and siShc-c in HaCaT cells), but not control siRNA, enhances the expression of Twist (E, F) and ZEB1 (G, H) mRNA, quantified by qRT-PCR and normalized against RPL19 mRNA, in the absence of or in response to 2 ng/ml TGF-β for 6 h. SB431542 prevents the enhanced Twist and ZEB1 mRNAs expression. Error bars indicate standard errors, based on three independent experiments. (I, J) Decreasing ShcA expression, upon transfection of two different siRNAs targeting ShcA, but not control siRNA, decreases E-cadherin (I) and enhances fibronectin (J) mRNA in NMuMG cells, in the absence of or in response to 2 ng/ml TGF-β for 72 h, and SB431542 inhibits the down-regulation of E-cadherin (I) and increase of fibronectin (J) mRNA expression. mRNA levels were quantified by qRT-PCR and normalized to RPL19 mRNA. Error bars indicate standard errors, based on three independent experiments. *, *p* < 0.05. Supplemental data are shown in S1 Data.
(TIF)

S6 Fig Corresponding to Fig 6. (A, B) Densitometry of the cell surface levels of TβRI (A) and TβRII (B), assessed by cell surface biotinylation, neutravidin adsorption and immunoblotting, of NMuMG cells transfected with control siRNA or ShcA siRNA (siShc-a), treated or not with TGF-β or SB431542 (as in Fig 6A). Error bars indicate standard errors based on three independent experiments. (C–E) Expression of TβRI (C), TβRII (D), and TGF-β1 (E) mRNAs in NMuMG cells, transfected with control siRNA or ShcA siRNA (siShc-a), treated or not with TGF-β or SB431542 (as in Fig 6A), was quantified by qRT-PCR and normalized against RPL19 mRNA. Error bars indicate standard errors, based on three independent experiments. (F) Active TGF-β released by NMuMG cells transfected with siRNA or ShcA siRNA (siShc-a) in serum-free DMEM for 16 h was measured using TMLC reporter cells. The active TGF-β measured without heating the media samples was compared with total released TGF-β, activated by heat treatment. (G, H) Increased p52ShcA expression results in decreased Smad3 activation, assessed by immunoblotting for pSmad3 (G), as shown in Fig 6B, and decreased Smad3D497E association with TβRI, assessed by immunoblotting for TβRI-associated Smad3 (H), as shown in Fig 6D. Densitometry compared their levels in NMuMG cells transfected with 0, 0.1, or 0.4 μg p52ShcA expression plasmid per well and treated with TGF-β (G) and in 293T cells cotransfected with 0, 0.1, or 0.4 μg p52ShcA expression plasmid per well and plasmids for TβRI and Smad3D407E and then treated with TGF-β (H). Error bars are based on three independent experiments. Supplemental data are shown in S1 Data.
(TIF)

S7 Fig Corresponding to Fig 7. (A–D). Densitometry of immunoblots showing that increased p52ShcA expression results in decreased association of β2 adaptin with TβRI (A), as shown in Fig 7A, and increased association of caveolin 1 with TβRI (C), as shown in Fig 7C, whereas increased Smad3 expression enhances the association of β2-adaptin with TβRI (B), as shown in Fig 7B, and decreases the interaction of caveolin 1 with TβRI (D), as shown in Fig 7D. Densitometry compared their levels in 293T cells transfected with 0, 0.1, or 0.4 μg p52ShcA-HA or Smad3-myc expression plasmid, as shown. Error bars are based on three independent experiments. (E, F) Densitometry of immunoblots of caveolin 1 (E) and β2-adaptin (F) that interacted with TβRI in NMuMG cells transfected with ShcA siRNA (siShc-a) or p52ShcA expression plasmid, treated or not with TGF-β, as shown in Fig 7C. Error bars are based on three independent experiments.
(TIF)

## Abbreviations

DAPI: 4’,6-diamidino-phenol-indole
EGF: epidermal growth factor
EMT: epithelial–mesenchymal transition
GAPDH: glyceraldehyde-3-phosphate dehydrogenase
HGF: hepatocyte growth factor
JNK: c-Jun N-terminal kinase
MAPK: mitogen-activated protein kinase
RTK: receptor tyrosine kinase
Ser: serine
siRNA: small interfering RNA
SiShc-a: ShcA siRNA
TGF-β: transforming growth factor-β
Thr: threonine
Tyr: tyrosine
