# Supplementary figures and images for: ShcA Protects against Epithelial–Mesenchymal Transition through Compartmentalized Inhibition of TGF-β-Induced Smad Activation
Source: PLoS Biol. 2015 Dec 17;13(12):e1002325. doi: 10.1371/journal.pbio.1002325 (PMC4682977; doi:10.1371/journal.pbio.1002325)

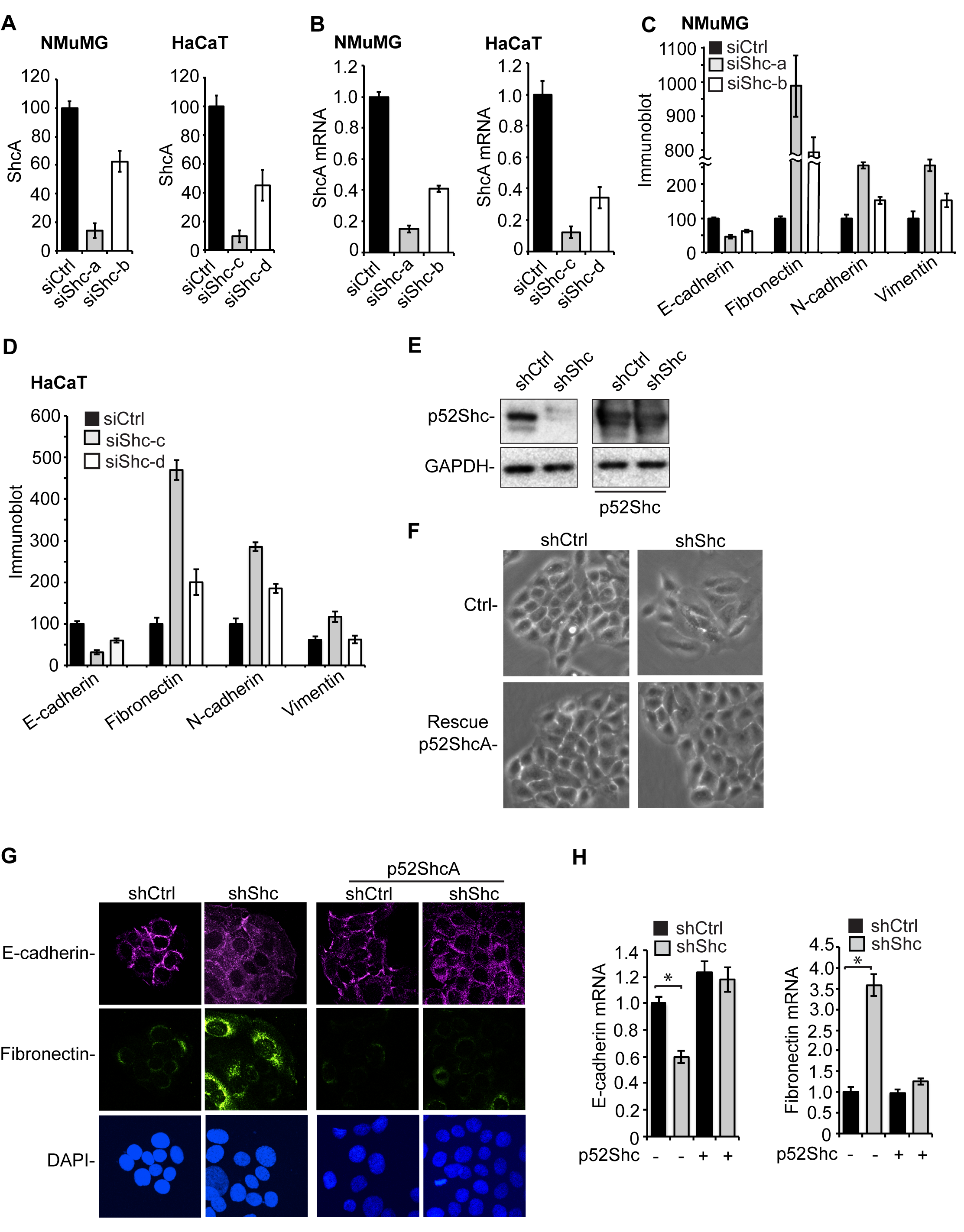

Supplement: S1 Fig — Decreased ShcA expression promotes EMT in NMuMG and HaCaT cells. (A) Selective silencing of ShcA expression in NMuMG and HaCaT cells using transfected siRNA, i.e., siShc-a and siShc-b for NMuMG cells, and siShc-c and siShc-d for HaCaT cells. NMuMG and HaCaT cells were transfected with control or ShcA siRNA, and the immunoblotted p52ShcA band was quantified by densitometry. The graphs show averaged values of three independent experiments, relative to p52ShcA of control siRNA cells. Error bars indicate standard errors, based on three independent experiments. (B) ShcA mRNA quantified by qRT-PCR and normalized against RPL19 mRNA in NMuMG cells or HaCaT cells transfected with control siRNA or ShcA siRNA, i.e., siShc-a and siShc-b for NMuMG cells, or siShc-c and siShc-d for HaCaT cells. Error bars indicate standard errors, based on three independent experiments. (C, D) Immunoblots of EMT marker expression, i.e., E-cadherin, fibronectin, N-cadherin, and vimentin, in NMuMG (C) and HaCaT (D) cells were quantified by densitometry. The graphs show averaged values of three independent experiments, relative to the marker expression in control siRNA cells. Error bars indicate standard errors, based on three independent experiments. (E) Compared to cells expressing a control shRNA, HaCaT cells infected to express shRNA targeting the 3’UTR of ShcA mRNA showed decreased ShcA expression, assessed by immunoblotting. Subsequent transfection with an siRNA-insensitive expression plasmid encoding p52ShcA resulted in increased p52ShcA expression. GAPDH immunoblotting was used as loading control. (F) Phase contrast microscopic images of HaCaT cells transfected with control or shRNA against the 3’UTR of ShcA mRNA and then rescued or not with a transfected p52ShcA plasmid. (G) Immunofluorecence detection of E-cadherin and fibronectin expression in HaCaT cells generated in (E) and shown in (F). (H) Expression of E-cadherin and fibronectin mRNAs in HaCaT cells generated in (E), quantified by qR [file pbio.1002325.s002.tif]

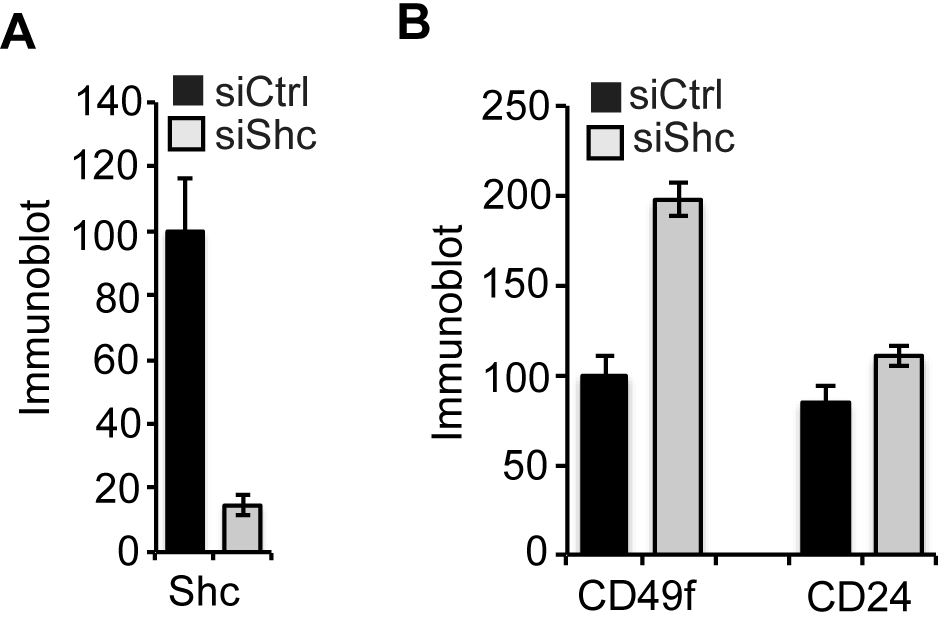

Supplement: S2 Fig — Densitometry analyses of (A) p52ShcA immunoblots of the NMuMG cells that were used for the zebrafish injection assays (Fig 2C–2E), and (B) CD24 and CD49f expression in the NMuMG cells that were used for mammosphere analyses (Fig 2F and 2G). Error bars indicate standard errors, based on three independent experiments. (TIF) [file pbio.1002325.s003.tif]

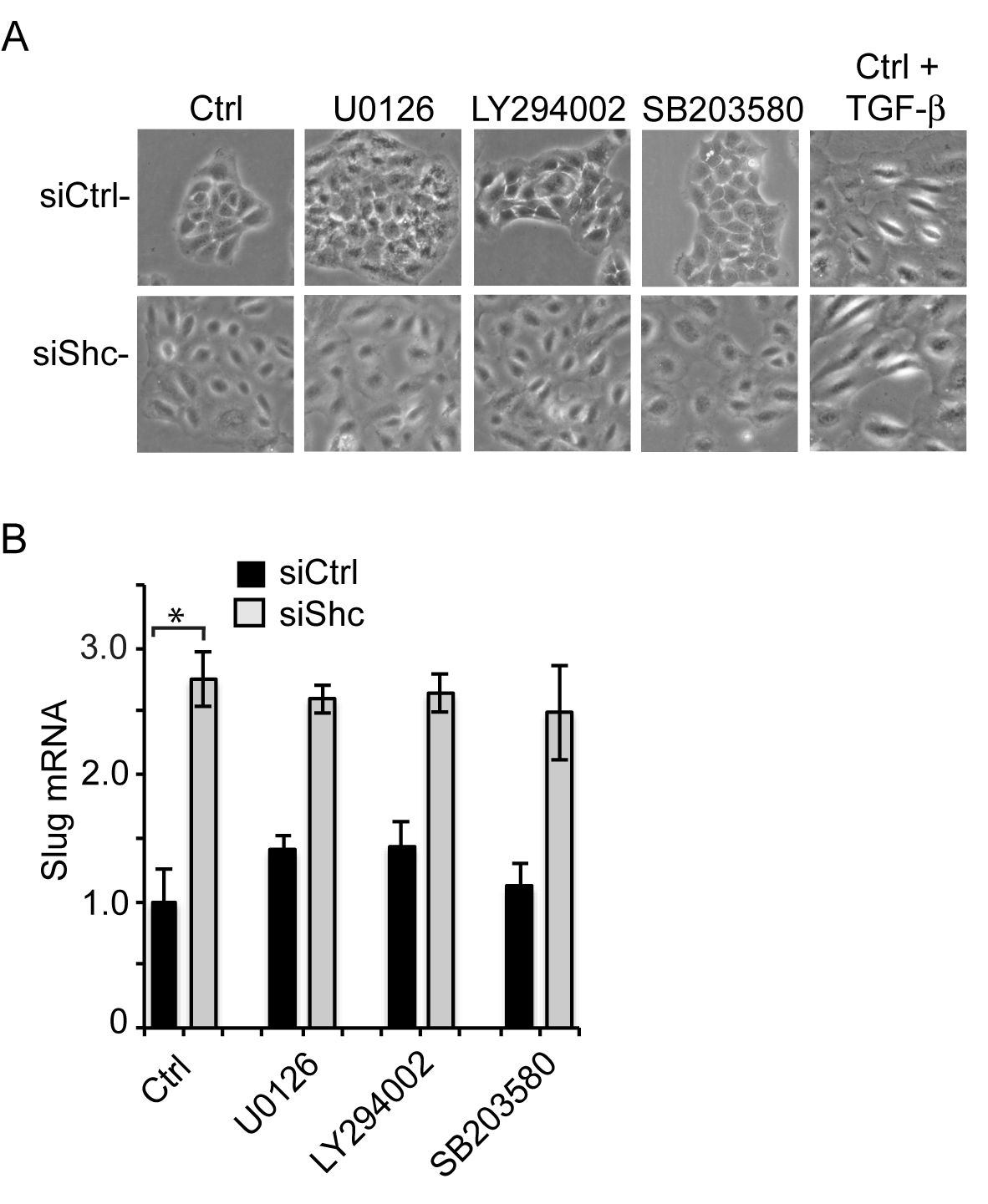

Supplement: S3 Fig — Effects of MEK1/2, PI3K, and p38 MAPK inhibition on the EMT phenotype of HaCaT cells with or without down-regulated ShcA expression. HaCaT cells transfected with control siRNA or ShcA siRNA (siShc-c) were treated or not with the MEK1/2 inhibitor U0126, the PI3K inhibitor LY294002, or the p38 MAPK inhibitor SB203580 for 36 h. In (A), the cell morphology was assessed by phase contrast microscopy, whereas in (B) Slug mRNA was quantified by qRT-PCR and normalized to RPL19 mRNA. The graphs show averaged values of three independent experiments, with error bars indicating standard errors, based on three experiments. Statistical analyses were performed using two-tailed two-sample unequal variance t test. *, p < 0.05. Supplemental data are shown in S1 Data. (TIF) [file pbio.1002325.s004.tif]

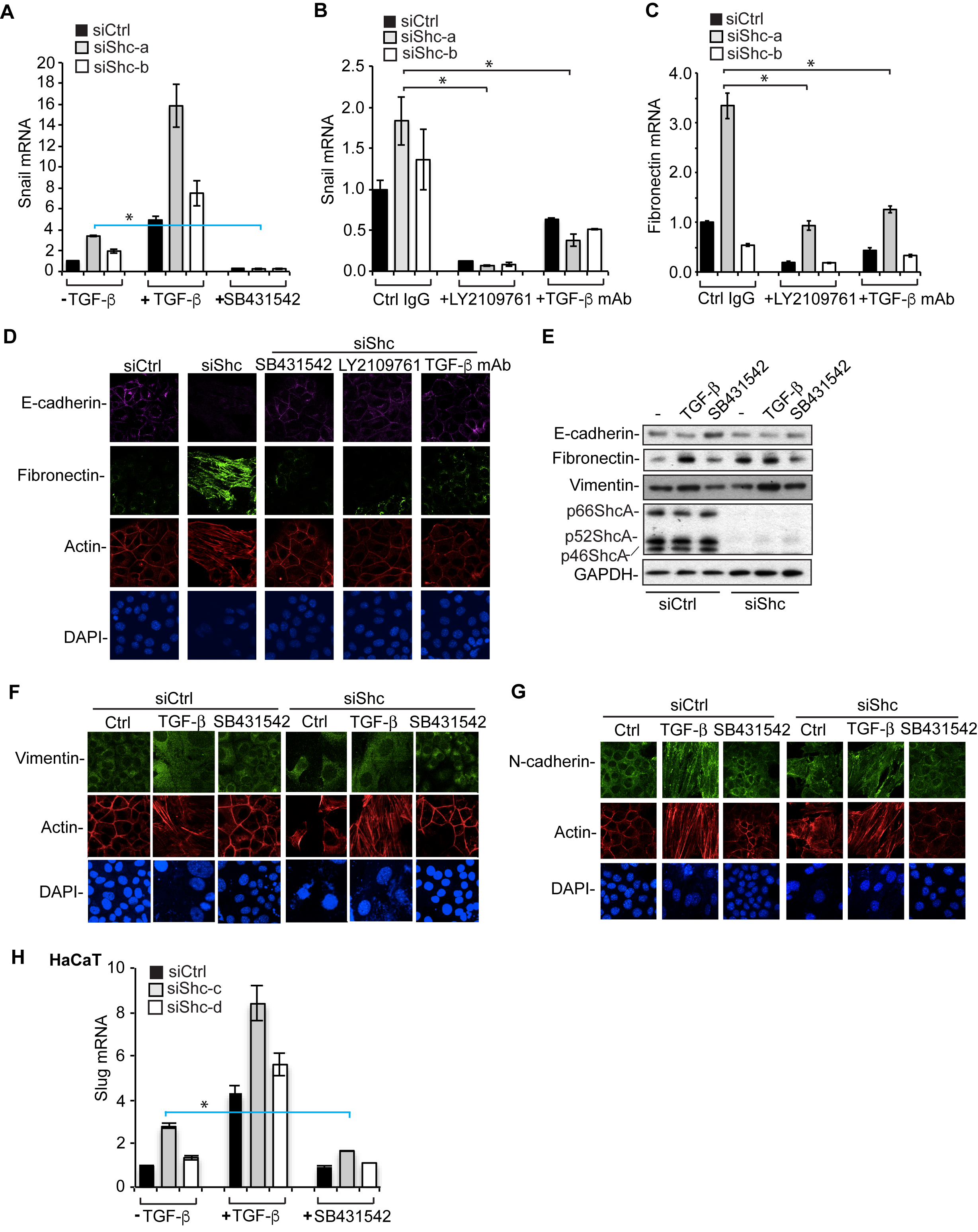

Supplement: S4 Fig — The TβRI kinase activity is required for EMT in epithelial cells with down-regulated ShcA expression. (A) Decreasing ShcA expression, upon transfection of two different siRNAs targeting ShcA, but not control siRNA, enhances Snail mRNA expression in NMuMG cells, in the absence of or in response to 2 ng/ml TGF-β for 6 h, and SB431542 prevents the enhanced Snail mRNA expression. mRNA levels were quantified by qRT-PCR and normalized to RPL19 mRNA. Error bars indicate standard errors, based on three independent experiments. (B, C) LY2109761 and TGF-β monoclonal antibody inhibit the increase in Snail mRNA (B) or fibronectin mRNA (C) in cells transfected with two different siRNAs targeting ShcA. (D) Effects of SB431542, LY2109761 or pan–anti-TGF-β monoclonal antibody on the expression of E-cadherin or fibronectin and actin organization in NMuMG cells transfected with control siRNA or ShcA siRNA (siShc-a), assessed by immunofluorescence. DAPI staining visualized the nuclei. (E) Effects of SB431542 on the expression of E-cadherin, fibronectin, and vimentin in NMuMG cells, transfected with control siRNA or ShcA siRNA (siShc-a), assessed by immunoblotting. GAPDH immunoblotting provided the loading control. (F, G) Effects of SB431542 on the expression of vimentin (F) and N-cadherin (G), and actin organization (F, G) in NMuMG cells transfected with control siRNA or ShcA siRNA (siShc-a), assessed by immunofluorescence. DAPI staining visualized the nuclei. (H) Decreasing ShcA expression, upon transfection of two different siRNAs targeting ShcA, but not control siRNA, enhances Slug mRNA expression in HaCaT cells, in the absence of or in response to 2 ng/ml TGF-β for 6 h, and SB431542 prevents the enhanced Slug mRNA expression. mRNA levels were quantified by qRT-PCR and normalized to RPL19 mRNA. Error bars indicate standard errors, based on three independent experiments. *, p < 0.05. All experiments were reproducibly repeated at least three times. Supplemental data are shown in S1 D [file pbio.1002325.s005.tif]

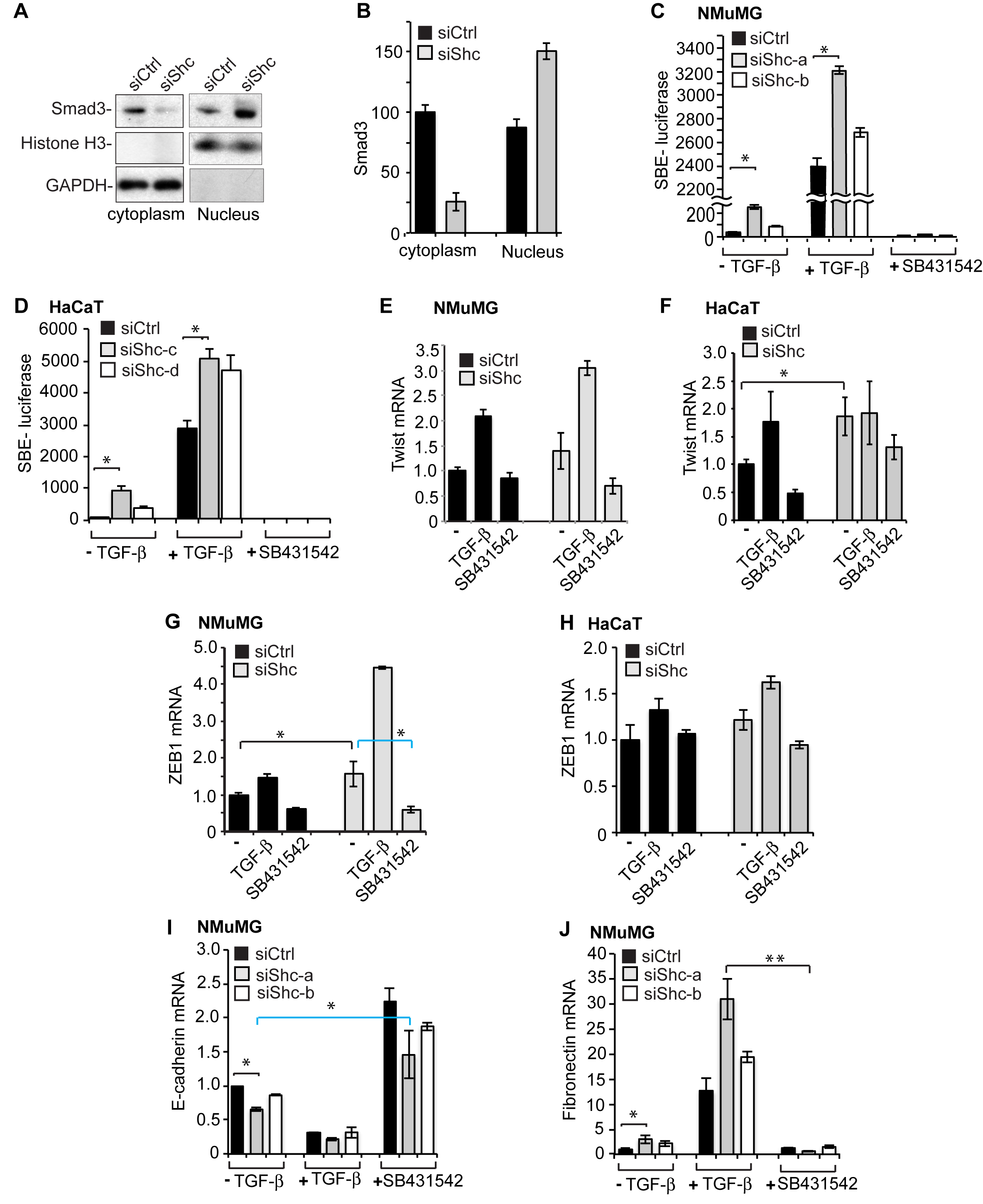

Supplement: S5 Fig — (A, B) Immunoblots of Smad3 in nuclear and cytoplasmic fractions of NMuMG cells transfected with ShcA siRNA (siShc-a) or control siRNA. Histone H3 and GAPDH serve as nuclear and cytoplasmic controls, respectively. Densitometric analyses of three independent experiments with standard errors are shown in B. (C, D) Decreasing ShcA expression, upon transfection of two different siRNAs targeting ShcA, but not control siRNA, enhances Smad3-mediated transcription, quantified by luciferase expression from a 4xSBE-luciferase reporter and normalized against the cotransfected Renilla-Lux reporter, in NMuMG (C) and HaCaT (D) cells, in the absence of or in response to 0.8 ng/ml TGF-β, or treated with SB431542 for 6 h. The TβRI kinase inhibitor SB431542 inhibits the luciferase expression. (E–H) Decreasing ShcA expression, upon transfection of NMuMG (E, G) and HaCaT (F, H) cells with ShcA siRNA (siShc-a in NMuMG and siShc-c in HaCaT cells), but not control siRNA, enhances the expression of Twist (E, F) and ZEB1 (G, H) mRNA, quantified by qRT-PCR and normalized against RPL19 mRNA, in the absence of or in response to 2 ng/ml TGF-β for 6 h. SB431542 prevents the enhanced Twist and ZEB1 mRNAs expression. Error bars indicate standard errors, based on three independent experiments. (I, J) Decreasing ShcA expression, upon transfection of two different siRNAs targeting ShcA, but not control siRNA, decreases E-cadherin (I) and enhances fibronectin (J) mRNA in NMuMG cells, in the absence of or in response to 2 ng/ml TGF-β for 72 h, and SB431542 inhibits the down-regulation of E-cadherin (I) and increase of fibronectin (J) mRNA expression. mRNA levels were quantified by qRT-PCR and normalized to RPL19 mRNA. Error bars indicate standard errors, based on three independent experiments. *, p < 0.05. Supplemental data are shown in S1 Data. (TIF) [file pbio.1002325.s006.tif]

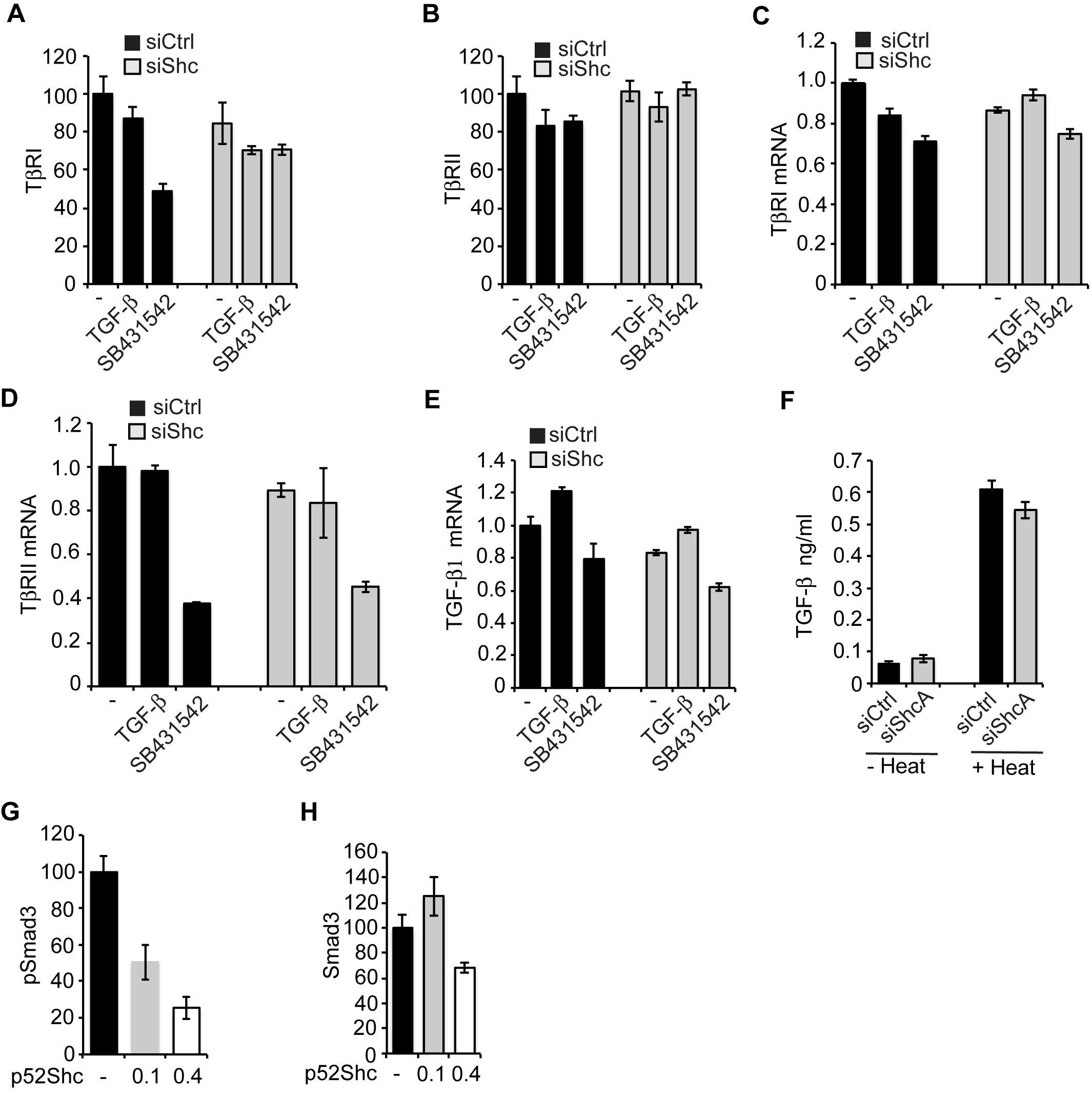

Supplement: S6 Fig — (A, B) Densitometry of the cell surface levels of TβRI (A) and TβRII (B), assessed by cell surface biotinylation, neutravidin adsorption and immunoblotting, of NMuMG cells transfected with control siRNA or ShcA siRNA (siShc-a), treated or not with TGF-β or SB431542 (as in Fig 6A). Error bars indicate standard errors based on three independent experiments. (C–E) Expression of TβRI (C), TβRII (D), and TGF-β1 (E) mRNAs in NMuMG cells, transfected with control siRNA or ShcA siRNA (siShc-a), treated or not with TGF-β or SB431542 (as in Fig 6A), was quantified by qRT-PCR and normalized against RPL19 mRNA. Error bars indicate standard errors, based on three independent experiments. (F) Active TGF-β released by NMuMG cells transfected with siRNA or ShcA siRNA (siShc-a) in serum-free DMEM for 16 h was measured using TMLC reporter cells. The active TGF-β measured without heating the media samples was compared with total released TGF-β, activated by heat treatment. (G, H) Increased p52ShcA expression results in decreased Smad3 activation, assessed by immunoblotting for pSmad3 (G), as shown in Fig 6B, and decreased Smad3D497E association with TβRI, assessed by immunoblotting for TβRI-associated Smad3 (H), as shown in Fig 6D. Densitometry compared their levels in NMuMG cells transfected with 0, 0.1, or 0.4 μg p52ShcA expression plasmid per well and treated with TGF-β (G) and in 293T cells cotransfected with 0, 0.1, or 0.4 μg p52ShcA expression plasmid per well and plasmids for TβRI and Smad3D407E and then treated with TGF-β (H). Error bars are based on three independent experiments. Supplemental data are shown in S1 Data. (TIF) [file pbio.1002325.s007.tif]

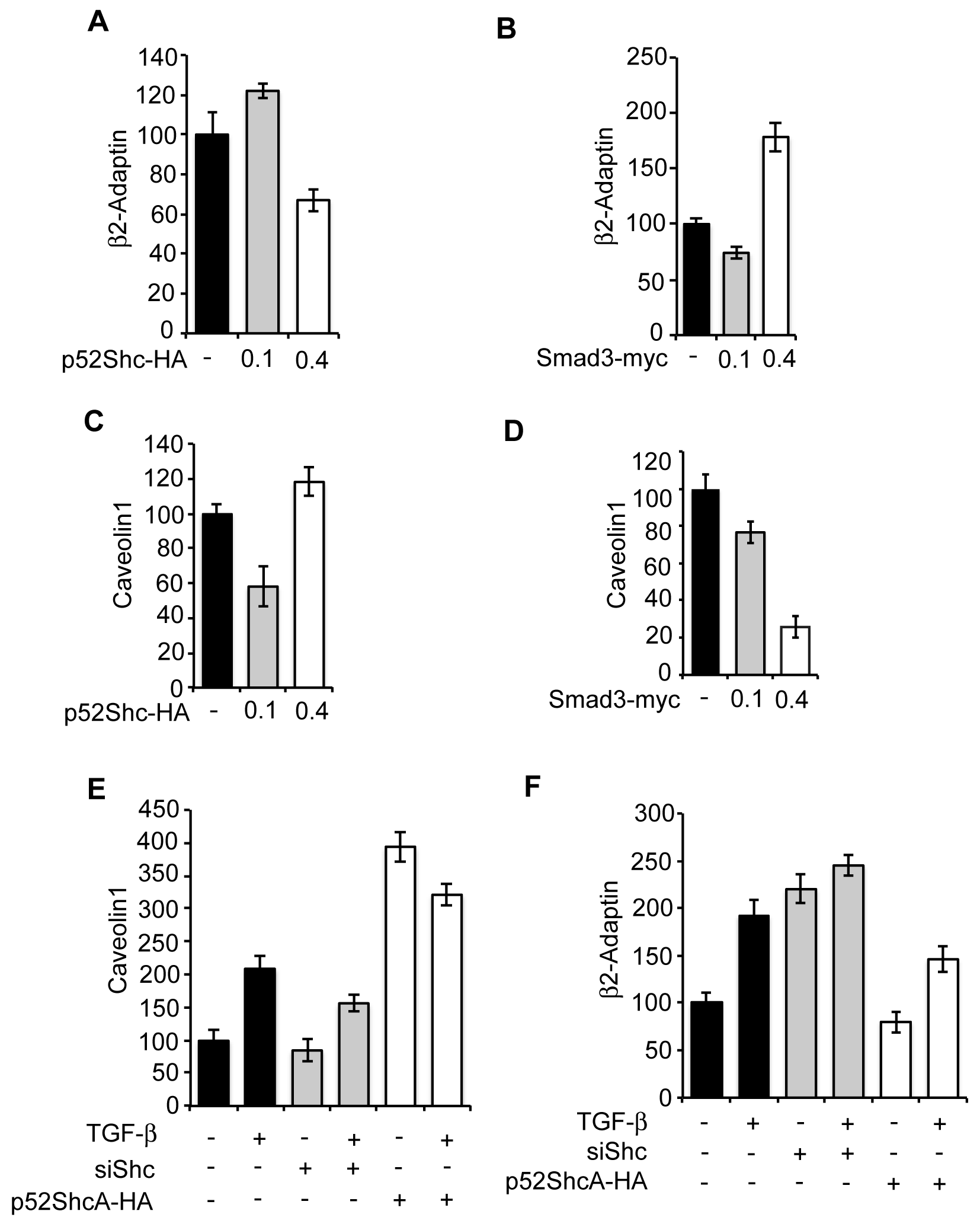

Supplement: S7 Fig — (A–D). Densitometry of immunoblots showing that increased p52ShcA expression results in decreased association of β2 adaptin with TβRI (A), as shown in Fig 7A, and increased association of caveolin 1 with TβRI (C), as shown in Fig 7C, whereas increased Smad3 expression enhances the association of β2-adaptin with TβRI (B), as shown in Fig 7B, and decreases the interaction of caveolin 1 with TβRI (D), as shown in Fig 7D. Densitometry compared their levels in 293T cells transfected with 0, 0.1, or 0.4 μg p52ShcA-HA or Smad3-myc expression plasmid, as shown. Error bars are based on three independent experiments. (E, F) Densitometry of immunoblots of caveolin 1 (E) and β2-adaptin (F) that interacted with TβRI in NMuMG cells transfected with ShcA siRNA (siShc-a) or p52ShcA expression plasmid, treated or not with TGF-β, as shown in Fig 7C. Error bars are based on three independent experiments. (TIF) [file pbio.1002325.s008.tif]
